# Optimized methods for preparation of 6^I^-(ω-sulfanyl-alkylene-sulfanyl)-β-cyclodextrin derivatives

**DOI:** 10.3762/bjoc.12.38

**Published:** 2016-02-24

**Authors:** Eva Bednářová, Simona Hybelbauerová, Jindřich Jindřich

**Affiliations:** 1 Department of Organic Chemistry, Faculty of Science, Charles University in Prague, Hlavova 8, 128 43, Prague 2, Czech Republic, Fax: +420 22195 1326; 2 Department of Teaching and Didactics of Chemistry, Faculty of Science, Charles University in Prague, Hlavova 8, 128 43, Prague 2, Czech Republic

**Keywords:** cyclodextrins, disulfides, monosubstituted derivatives, thiols

## Abstract

A general high-yielding method for the preparation of monosubstituted β-cyclodextrin derivatives which have attached a thiol group in position 6 is described. The thiol group is attached through linkers of different lengths and repeating units (ethylene glycol or methylene). The target compounds were characterized by IR, MS and NMR spectra. A simple method for their complete conversion to the corresponding disulfides as well as a method for the reduction of the disulfides back to the thiols is presented. Both, thiols and disulfides are derivatives usable for well-defined covalent attachment of cyclodextrin to gold or polydopamine-coated solid surfaces.

## Introduction

Cyclodextrins (CDs) [1], cyclic oligomers of α-D-glucopyranose, are used for their ability to form supramolecular inclusion complexes with a wide range of guest molecules [2]. From the native CDs (α-CD, β-CD, and γ-CD containing 6, 7, and 8 glucose units, respectively) the β-CD is studied the most due to its lowest price and the highest guest binding ability. A large number of CD derivatives [3] which have been prepared up to now can be divided into several groups according to their intended use. The largest group is the one containing CD derivatives with modified complexation properties, used mainly as solubilizers and/or stabilizers in pharmaceutical, cosmetic, agricultural and food industries [4]. These derivatives are usually mixtures of randomly substituted CDs. On the other hand, CD derivatives which are well-defined chemical individuals are well suited for separation science [5], chemosensors [6] or drug delivery [7] applications. These applications often involve a modification of solid surfaces by CDs. Among the solid surfaces, to which CDs were attached, are sorbents for separation techniques or waste water treatment [8] or nanoparticles [6].

In our work, we focused on the preparation of sulfanyl-group-containing CD derivatives – 6^I^-deoxy-6^I^-(ω-sulfanyl-alkylene-sulfanyl)-β-cyclodextrins (β-CD-S-X-SH) – which can be attached to a gold surface [9–10] or a polydopamine-coated surface [11]. It has been already described that the surface coverage of self-assembled monolayers of CD derivatives on gold depends substantially on the linker between the sulfanyl group and the CD. Derivatives with sulfanyl groups connected directly to the CD gave much lower surface coverage [12] than the derivatives containing just one sulfanyl group connected by a longer linker [13]. Therefore, we decided to prepare a series of CD sulfanyl derivatives with different lengths of the linker connecting the CD and the sulfanyl group. These derivatives are needed for two projects which both will study the influence of the linker length on the properties of the whole system. The first project will study the sensitivity of quartz crystal microbalances with a CD-modified gold sensor [14]; the second will study photodynamic sensitizers included into CDs attached on the surface of polydopamine-covered nanowires [15]. Syntheses of several sulfanyl group-containing CD derivatives suitable for this purpose were published. The first method [16] is using a direct substitution of 6^I^-*O*-(*p*-toluenesulfonyl)-β-CD with dithiols. We also intended to use this method, but the described procedure gives only yields around 20%. The second method [13] is using a disulfide of mercaptopropionic acid which is coupled by an amide bond to two molecules of 6^I^-amino-β-CD to form a stable CD disulfide derivative. This disulfide can be used directly for functionalization of a gold surface, but its reduction to thiol was not described. In any case, the amide-containing derivative might not be stable enough under basic conditions needed for the polydopamine derivatization by the thiol. Besides, the common problem with the preparation of this type of sulfanyl derivatives – formation of disulfide byproducts – was not addressed in the literature at all. Therefore, we decided to develop more general procedures for the preparation of such CD derivatives and also a method for controlled preparation of corresponding disulfides, which are much more stable alternatives to the thiols for a longer storage. That is why a method to recover the thiols from the disulfides was also studied.

## Results and Discussion

For the preparation of the starting compound for all syntheses – Ts-β-CD (**1**) – we recently described [17] a modification of one of the commonly used method [18]. The modification consisted in the repeated recrystallization from 50% water–methanol which yielded a very pure product with no interfering impurities (di- or tri-Ts-β-CD) or unreacted β-CD.

**Scheme 1:**
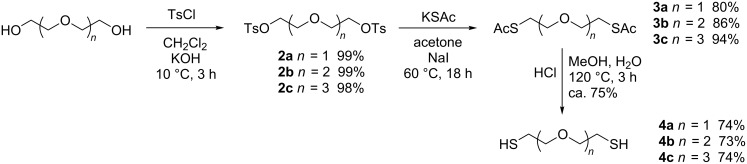
Synthesis of oligoethylene glycol dithiols.

The terminal sulfanyl groups were introduced to the oligoethylene glycol linkers using standard chemical transformations (Scheme 1). Oligoethylene glycol ditosylates **2a**–**c** [19] were converted to *S*-acetyl dithiols **3a**–**c** [20] and deacetylated to the oligoethylene glycol dithiols **4a**–**c** using a procedure published for similar thiols [21]. Compounds **4** can be easily oxidized to disulfides; therefore their preparation and purification must be performed under inert atmosphere.

**Scheme 2:**
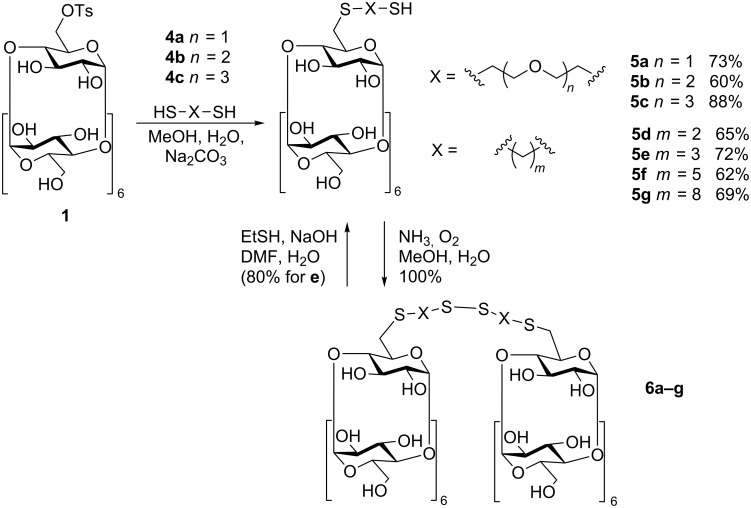
Synthesis of β-cyclodextrinthiols and -disulfides.

Then we optimized methods for the preparation of β-CD-S-X-SH derivatives (**5a**–**g**, Scheme 2*)* from Ts-β-CD for two types of compounds in which X is either a pure alkylene group –(CH_2_)*_m_*– (*m* = 2, 3, 5, 8) or the oligoethylene glycol group –CH_2_(CH_2_OCH_2_)*_n_*CH_2_– (*n* = 1, 2, 3). In addition, general high yielding methods for conversion of the prepared CD-thiols to CD disulfides (**6a**–**g**) by air oxidation and the CD disulfides back to CD-thiols by reduction with ethanethiol were developed.

At first, we tried to reproduce the synthesis of the compound **5g** (Scheme 2) according to the published procedure [12]. Product **5g** was detected in the reaction mixture by MS but to isolate it in a sufficient yield was difficult. The main byproduct was the corresponding disulfide **6g** which is formed from **5g** by air oxidation. Moreover, the obtained alkylene derivative **5g** had a low solubility in water and water/alcohol mixtures what represented quite an obstacle for the intended use – derivatization of solid surfaces by treatment with the water solution of the CD derivative.

Therefore, we decided also to prepare the derivatives containing the more hydrophilic and flexible oligoethylene glycol linker, which have been successfully applied in several studies to position the CD units at a sufficient distance from the solid surface. These linkers also have the advantage, compared to the aliphatic chain linkers, in a lower tendency to form an inclusion complex with β-CD [22], i.e., a suitable accessibility of the cavity should be kept, and further inclusion complexes could be formed with the guests.

Strict inert conditions are also required for the next synthetic step – preparation of compounds **5**. The air had to be removed from the reaction mixture by bubbling argon through the sonicated solution. The ideal solvent mixture for this reaction, which dissolves both reactants, proved to be water/methanol 1:1 in which the reaction reached a complete conversion of the starting Ts-β-CD in 20 h. The crucial step of the work-up was the acidification of the basic reaction mixture and the extraction of the unreacted dithiols with chloroform. The prepared CD-thiols form disulfides very quickly under basic conditions but are reasonably stable under neutral and acidic conditions. Therefore, the final chromatographic separation could not be done in the common elution mixture (propan-1-ol/water/ammonia) but the neutral solvent mixture (butan-1-ol/ethanol/water) was used instead. This optimized procedure gave the target compounds **5a–g** in 60–90% yield, and the unreacted dithiols could be easily recovered. Oxidation products of compounds **5a**–**g** (disulfides **6a**–**g)** are quite stable compounds and were prepared in quantitative yield just by bubbling air through the water/methanol solution of the thiol which was basified by addition of aqueous ammonia. The disulfides **6** can be used, as well as the thiols, for the attachment of the cyclodextrin cavity to a gold surface. So they can be used instead of the thiols in applications where well defined starting compounds are needed. On the other hand, for applications where only the thiol can be used (like attachment to polydopamine-coated surfaces), and the pure compound is often needed, the disulfide can be prepared in larger amounts and used for a long term storage. The corresponding pure thiol can be then prepared when needed by a simple one step high yielding procedure developed for compound **6e** (Scheme 2), which does not require any chromatographic separation and uses ethanethiol in basic solution as the reducing agent. The separation of the thiol product consists just of its precipitation with acetone from the reaction mixture. This separation method is very efficient and is used very often for separation of monosubstituted CD derivatives. The same reaction conditions, as for compound **6e**, were used for the reduction of all disulfides **6** on TLC scale. The TLC proved a conversion to the thiol for all of them. The only compound which was not completely converted under the given conditions was **6g**, most probably due to its low solubility.

## Conclusion

To conclude, we addressed problems commonly encountered during the syntheses of thiol-group-containing cyclodextrin derivatives and presented reproducible methods for their elimination. The main problem – formation of the corresponding disulfides by air oxidation – could be suppressed substantially by using an inert atmosphere and performing work-up under acidic conditions. Moreover, the often unwanted and much more stable disulfide byproducts can be obtained from the thiols in quantitative yields and can be used instead of them in some applications. Disulfides can also be used for a long-term storage and the corresponding thiol can be prepared in one simple synthetic step and isolated by precipitation.

## Supporting Information

File 1 General experimental procedures, instruments, materials. Detailed experimental procedures and characterization data for newly prepared compounds. ^1^H and ^13^C NMR spectra of prepared compounds.
