# Supplementary material for: Optimized methods for preparation of 6I-(ω-sulfanyl-alkylene-sulfanyl)-β-cyclodextrin derivatives
Source: Beilstein J Org Chem. 2016 Feb 24;12:349–52. doi: 10.3762/bjoc.12.38 (PMC4778511; doi:10.3762/bjoc.12.38)

Supporting Information

for

Optimized methods for preparation of 6^{I}-(ω-sulfanyl-alkylene-sulfanyl)-β-cyclodextrin derivatives

Eva Bednářová[1], Simona Hybelbauerová[2], Jindřich Jindřich[1*]

Address: [1]Department of Organic Chemistry, Faculty of Science, Charles University in Prague, Hlavova 8, 128 43, Prague 2, Czech Republic, Fax: +420 22195 1326 and [2]Department of Teaching and Didactics of Chemistry, Faculty of Science, Charles University in Prague, Hlavova 8, 128 43, Prague 2, Czech Republic

E-mail: Jindřich Jindřich - jindrich@natur.cuni.cz

*Corresponding author

General experimental procedures, instruments, materials. Detailed experimental procedures and characterization data for newly prepared compounds. ^{1}H and ^{13}C NMR spectra of prepared compounds

Contents

General information s3

General procedure for the connection of dithiol to β-cyclodextrin (GP1) s4

6^{I}-Deoxy-6^{I}-((5-sulfanyl-3-oxapentane-1-yl)sulfanyl)-β-CD (**5a**) s4

6^{I}-Deoxy-6^{I}-((8-sulfanyl-3,6-dioxaoctane-1-yl)sulfanyl)-β-CD (**5b**) s4

6^{I}-Deoxy-6^{I}-((11-sulfanyl-3,6,9-trioxaundecane-1-yl)sulfanyl)-β-CD (**5c**) s5

6^{I}-Deoxy-6^{I}-((2-sulfanylethane-1-yl)sulfanyl)-β-CD (**5d**) s5

6^{I}-Deoxy-6^{I}-((3-sulfanylpropane-1-yl)sulfanyl)-β-CD (**5e**) s6

6^{I}-Deoxy-6^{I}-((5-sulfanylpentane-1-yl)sulfanyl)-β-CD (**5f**) s6

6^{I}-Deoxy-6^{I}-((8-sulfanyloktane-1-yl)sulfanyl)-β-CD (**5g**) s7

Procedure for regeneration of unreacted dithiol from the reaction mixture s7

General procedure for oxidation of cyclodextrin thiols into corresponding disulfides (GP2) .. s7

Disulfide **6a** s8

Disulfide **6b** s8

Disulfide **6c** s8

Disulfide **6d** s9

Disulfide **6e** s9

Disulfide **6f** s9
Disulfide **6g** s10
Procedure for reduction of cyclodextrin disulfide to the corresponding thiol s10
General procedure of ditosylation of oligo ethylene glycols (GP3) s11
3-Oxapentan-1,5-diyl-bis(*p*-toluensulfonate) (**2a**) s11
3,6-Dioxaoctane-1,8-diyl-bis(*p*-toluensulfonate) (**2b**) s11
3,6,9-Trioxaundecane-1,11-diyl-bis(*p*-toluensulfonate) (**2c**) s11
General procedure for bis(thioacetylation) ditosylated oligo ethylene glycols (GP4) s11
S,S'-(3-Oxapentane-1,5-diyl)-bis(thioacetate) (**3a**) s12
S,S'-(3,6-Dioxaoctane-1,8-diyl)-bis(thioacetate) (**3b**) s12
S,S'-(3,6,9-Trioxaundecane-1,11-diyl)-bis(thioacetate) (**3c**) s12
General procedure for deacetylation of bis(thioacetylated) oligo ethylene glycols (GP5) s12
3-Oxapentane-1,5-dithiol (**4a**) s13
3,6-Dioxaoctane-1,8-dithiol (**4b**) s13
3,6,9-Trioxaundecane-1,11-dithiol (**4c**) s13
References s13
^{1}H and ^{13}C NMR spectra s14
6^{I}-Deoxy-6^{I}-((5-sulfanyl-3-oxapentane-1-yl)sulfanyl)-β-CD (**5a**) s14
6^{I}-Deoxy-6^{I}-((8-sulfanyl-3,6-dioxaoctane-1-yl)sulfanyl)-β-CD (**5b**) s15
6^{I}-Deoxy-6^{I}-((11-sulfanyl-3,6,9-trioxaundecane-1-yl)sulfanyl)-β-CD (**5c**) s16
6^{I}-Deoxy-6^{I}-((2-sulfanylethane-1-yl)sulfanyl)-β-CD (**5d**) s17
6^{I}-Deoxy-6^{I}-((3-sulfanylpropane-1-yl)sulfanyl)-β-CD (**5e**) s18
6^{I}-Deoxy-6^{I}-((5-sulfanylpentane-1-yl)sulfanyl)-β-CD (**5f**) s19
6^{I}-Deoxy-6^{I}-((8-sulfanyloktane-1-yl)sulfanyl)-β-CD (**5g**) s20
Disulfide **6a** s21
Disulfide **6b** s22
Disulfide **6c** s23
Disulfide **6d** s24
Disulfide **6e** s25
Disulfide **6f** s26
Disulfide **6g** s27

General information

Chemicals and solvents for reactions were purchased from Sigma-Aldrich (reagent grade) if not otherwise noted. β-CD (97%) was purchased from WAKO chemicals. Solvents were distilled prior to use if not otherwise noted. Ethanol (96%) (EtOH) and butan-1-ol (99.5%) (BuOH) for chromatography were purchased from PENTA and used without further purification. Ratio of solvents in elution mixtures is given as volume/volume. Argon was used as an inert gas.

Silica gel 60 (0.040–0.063 mm) was used for column chromatography, TLC was performed on aluminum sheets with a layer of Silica gel 60 F_{254}, both purchased from MERCK. Spots on TLC plates were detected by using an UV lamp (*λ* = 254 nm) or by derivatization by potassium permanganate (basic aqueous solution) or sulfuric acid (50% aqueous solution).

NMR spectra were recorded on Varian VNMRS 300 ($\nu(^{1}H)$ = 299.94 MHz, $\nu(^{13}C)$ = 75.43 MHz) (for nonCD derivatives) or on Bruker Avance III (*v*(1H) = 600.17 MHz, *v*(13C) = 150.04 MHz) (for CD derivatives) in deuterated solvents and referenced to residual solvent peak. Chemical shifts are given in δ-scale, coupling constants *J* are given in Hz. Numbering of atoms for NMR spectra transcription was done according to structures on Figure SI-1.

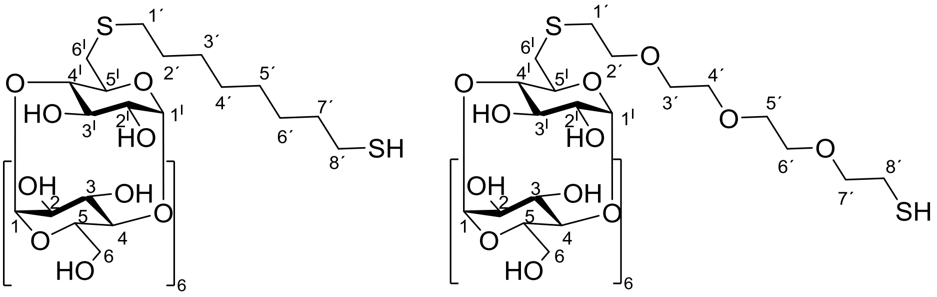

Figure SI-1: Numbering of atoms in cyclodextrin derivative structures

NMR characterization was done by using ^{1}H, ^{13}C, APT and 2D NMR experiments such as COSY, HSQC and HMBC. Because the measurement of the NMR spectra of the CDs was a time consuming process, disulfide derivatives from thiols were formed during this period by oxidation on air. This process was probably even increased by using DMSO as deuterated solvent. This is the reason why in some spectra of the thiols also peaks of disulfides can be seen. The amount of non-oxidized product is given immediately after the NMR spectra transcription.

Infrared spectra were recorded in KBr and measured on a Hermo Nicolet AVATAR 370 FT-IR spectrometer. Mass spectra were measured on a Bruker ESQUIRE 3000 ES-ion trap spectrometer.

General procedure for the connection of dithiol to β-cyclodextrin (GP1)

Tosyl cyclodextrin **1** (300 mg, 0.23 mmol) was suspended in a mixture of methanol (15 mL), water (15 mL), and sodium carbonate (62 mg, 2.5 equiv) under an inert atmosphere. To remove dissolved oxygen, argon was bubbled through this mixture in advance (2 minutes), exposed to ultrasound (10 minutes) and bubbled by argon again (10 minutes). Then the dithiol (9.5 equiv) was added to the solution, which was then stirred under a condenser and an inert atmosphere at 50 °C for 20 hours. The reaction process was monitored by TLC (BuOH/EtOH/H_2O 5/4/4, detection by H_2SO_4). After cooling to room temperature, the reaction mixture was neutralized by addition of hydrochloric acid (5% aqueous solution). After dilution of the reaction mixture to double volume by addition of water, unreacted dithiol was removed by extraction with chloroform (3 × 1:1). The solvents of the water layer were evaporated under reduced pressure. The solid residue was adsorbed on silica gel (10 × weight of the residue) and purified by column chromatography on silica gel (200 × weight of the residue).

6^{I}-Deoxy-6^{I}-((5-sulfanyl-3-oxapentane-1-yl)sulfanyl)-β-CD (5a)

By the general procedure GP1 233 mg of compound **5a** was obtained from cyclodextrin derivative **1** (330 mg, 0.26 mmol) and dithiol 4**a** in the form of a white solid in 73% yield. TLC – R_f = 0.53. Column chromatography – BuOH/EtOH/H_2O 5/2/2. ^{1}H NMR (600 MHz, d_6-DMSO): δ = 5.80–5.64 (m, 14 H, 7 × OH-2, 7 × OH-3), 4.86 (d, J = 3.4 Hz, 1 H, H-1), 4.85–4.80 (m, 6 H, 6 × H-1), 4.48 (t, J = 5.4 Hz, 1 H, OH-6), 4.46–4.41 (m, 5 H, 5 × OH-6), 3.81–3.76 (m, 2 H, H-5^{I}, H-6), 3.71–3.53 (m, 26 H, 7 × H-3, 2 × H-4, 6 × H-5, 11 × H-6), 3.52 (t, J = 6.7 Hz, 2 H, 2 × H-2´), 3.48 (t, J = 6.6 Hz, 2 H, 2 × H-3´), 3.41–3.27 (m, 7 × H-2, 5 × H-4 + H_2O), 3.04–2.99 (m, 1 H, H-6^{I}), 2.76 (dd, J = 13.8, 7.2 Hz, 1 H, H-6^{I}), 2.74–2.66 (m, 2 H, 2 × H-1´), 2.63–2.57 (m, 2 H, H-4´), 2.24 (t, J = 8.1 Hz, 1 H, SH) ppm. ^{13}C NMR (150 MHz, d_6-DMSO): δ = 102.29 (C-1), 101.97–101.94 (5 × C-1), 101.64 (C-1), 84.87 (C-4^{I}), 81.55–81.37 (6 × C-4), 73.07–71.98 (7 × C-2, 7 × C-3, 6 × C-5), 71.76 (C-3´), 71.27 (C-5^{I}), 69.63 (C-2´), 59.90 (6 × C-6), 33.44 (C-6^{I}), 31.93 (C-1´), 23.45 (C-4´) ppm. Content of the non-oxidized product in the NMR spectrum according to the ^{1}H NMR: 99%. IR (drift KBr): υ = 3312, 1162, 1102, 1078, 1036. MS (ESI): $[M+Na]^+$ = 1277.5. For $C_{46}H_{78}NaO_{35}S_2$ calculated 1277.3663.

6^{I}-Deoxy-6^{I}-((8-sulfanyl-3,6-dioxaoctane-1-yl)sulfanyl)-β-CD (5b)

By the general procedure GP1 180 mg of compound **5b** was obtained from cyclodextrin derivative **1** (300 mg, 0.23 mmol) and dithiol 4**b** in the form of a white solid in 60% yield. TLC – R_f = 0.56. Column chromatography –

BuOH/EtOH/H_2O 5/2/2. ^1H NMR (600 MHz, d_6-DMSO): δ = 5.87–5.65 (m, 14 H, 7 × OH-2, 7 × OH-3), 4.86 (d, J = 3.6 Hz, 1 H, H-1), 4.85–4.80 (m, 6 H, 6 × H-1), 4.50–4.40 (m, 6 H, 6 × OH-6), 3.82–3.73 (m, 2 H, H-5^I, H-6), 3.71–3.44 (m, 34 H, 7 × H-3, 2 × H-4, 6 × H-5, 11 × H-6, 2 × H-2′, 2 × H-3′, 2 × H-4′, 2 × H-5′), 3.43–3.23 (m, 7 × H-2, 5 × H-4 + H_2O), 3.04–2.98 (m, 1 H, H-6^I), 2.82–2.74 (m, 1 H, H-6^I), 2.74–2.66 (m, 2 H, 2 × H-1′), 2.61 (t, J = 6.5, 2 H, 2 × H-6′), 2.30–2.20 (br s, 1 H, SH) ppm. ^{13}C NMR (150 MHz, d_6-DMSO): δ = 102.29 (C-1), 101.96 (5 × C-1), 101.70 (C-1), 84.72 (C-4^I), 81.55–81.50 (6 × C-4), 73.01–71.99 (7 × C-2, 7 × C-3, 6 × C-5), 71.32 (C-5^I), 70.07 (C-5′), 70.01 (C-2′), 69.54–69.37 (C-3′, C-4′), 59.92 (6 × C-6), 33.27 (C-6^I), 31.86 (C-1′), 23.45 (C-6′) ppm, in agreement with the literature[5]. Content of the non-oxidized product in the NMR spectrum according to the ^1H NMR: 74%. IR (drift KBr): υ = 1153, 1078, 1027, 576 cm^{-1}. MS (ESI): $[M+Na]^+$ = 1321.5. For $C_{48}H_{82}NaO_{36}S_2$ calculated 1321.3925.

6^I-Deoxy-6^I-((11-sulfanyl-3,6,9-trioxaundecane-1-yl)sulfanyl)-β-CD (5c)

By the general procedure GP1 278 mg of compound **5c** was obtained from cyclodextrin derivative **1** (302 mg, 0.23 mmol) and dithiol 4**c** in the form of a white solid in 88% yield. TLC – R_f = 0.54. Column chromatography – BuOH/EtOH/H_2O 5/2/2. ^1H NMR (600 MHz, d_6-DMSO): δ = 5.80–5.66 (m, 14 H, 7 × OH-2, 7 × OH-3), 4.86 (d, J = 3.6 Hz, 1 H, H-1), 4.85–4.81 (m, 6 H, 6 × H-1), 4.49–4.42 (m, 6 H, 6 × OH-6), 3.80–3.72 (m, 2 H, H-5^I, H-6), 3.71–3.53 (m, 38 H, 7 × H-3, 2 × H-4, 6 × H-5, 11 × H-6, 2 × H-2′, 2 × H-3′, 2 × H-4′, 2 × H-5′, 2 × H-6′, 2 × H-7′), 3.41–3.26 (m, 7 × H-2, 5 × H-4 + H_2O), 3.04–2.98 (m, 1 H, H-6^I), 2.81–2.75 (m, 1 H, H-6^I), 2.75–2.66 (m, 2 H, 2 × H-1′) 2.62 (dt, J = 8.0, 6.6 Hz, 2 H, 2 × H-8′), 2.26 (t, J = 6.4 Hz, 1 H, SH) ppm. ^{13}C NMR (150 MHz, d_6-DMSO): δ = 102.29 (C-1), 101.97 (5 × C-1), 101.67 (C-1), 84.89 (C-4^I), 81.56–81.40 (6 × C-4), 73.06–71.99 (7 × C-2, 7 × C-3, 6 × C-5), 71.36 (C-5^I), 70.05–69.41 (C-2′, C-3′, C-4′, C-5′, C-6′), 68.62 (C-7′), 59.92 (6 × C-6), 33.33 (C-6^I), 31.88 (C-1′), 23.42 (C-8′) ppm. Content of the non-oxidized product in the NMR spectrum according to the ^1H NMR: 76%. IR (drift KBr): υ = 1156, 1078, 1033, 579 cm^{-1} MS (ESI): $[M+Na]^+$ = 1365.6. For $C_{50}H_{86}NaO_{37}S_2$ calculated 1365.4187.

6^I-Deoxy-6^I-((2-sulfanylethane-1-yl)sulfanyl)-β-CD (5d)

By the general procedure GP1 182 mg of compound **5d** was obtained from cyclodextrin derivative **1** (298 mg, 0.23 mmol) and ethane-1,2-dithiol in the form of a white solid in 65% yield. TLC – R_f = 0.50. Column chromatography – BuOH/EtOH/H_2O 5/2/2 (2000 ml) then 5/4/4. ^1H NMR (600 MHz, d_6-DMSO): δ = 5.85–5.64 (m, 14 H, 7 × OH-2, 7 × OH-3), 4.87 (d, J = 3.7 Hz, 1 H, H-1), 4.85–4.81 (m, 6 H, 6 × H-1), 4.53 (t, J = 5.2 Hz, 1 H, OH-6), 4.47–4.41 (m, 4 H, 4 × OH-6), 4.31 (t, J = 5.1 Hz, 1 H, OH-6), 3.82–3.73 (m, 2 H, H-5^I, H-6), 3.70–3.52 (m, 26 H, 7 × H-3, 2 × H-4, 6 × H-5, 11 ×

H-6), 3.40–3.25 (m, 7 × H-2, 5 × H-4 + H_2O), 3.04–2.98 (m, 1 H, H-6^I), 2.78–2.69 (m, 3 H, H-6^I, 2 × H-1′), 2.66–2.59 (m, 2 H, 2 × H-2′) ppm. ^{13}C NMR (150 MHz, d_6-DMSO): δ = 102.31 (C-1), 101.97–101.95 (5 × C-1), 101.58 (C-1), 84.98 (C-4^I), 81.62–81.33 (6 × C-4), 73.06–71.99 (7 × C-2, 7 × C-3, 6 × C-5), 71.09 (C-5^I), 60.36–59.80 (6 × C-6), 34.68 (C-1′), 32.99 (C-6^I), 24.29 (C-2′) ppm. Content of the non-oxidized product in the NMR spectrum according to the ^{1}H NMR: 92%. IR (drift KBr): υ = 3309, 1156, 1075, 1036 cm^{-1}. MS (ESI): $[M+Na]^+$ = 1232.4. For $C_{44}H_{74}NaO_{34}S_2$ calculated 1233.3401.

6^I-Deoxy-6^I-((3-sulfanylpropane-1-yl)sulfanyl)-β-CD (5e)

By the general procedure GP1 275 mg of compound **5e** was obtained from cyclodextrin derivative **1** (400 mg, 0.31 mmol) and propane-1,3-dithiol in the form of a white solid in 72% yield. TLC – R_f = 0.55. Column chromatography – BuOH/EtOH/H_2O = 5/2/2. ^{1}H NMR (600 MHz, d_6-DMSO): δ = 6.01–5.68 (m, 14 H, 7 × OH-2, 7 × OH-3), 4.86 (d, J = 3.7 Hz, 1 H, H-1), 4.85–4.79 (m, 6 H, 6 × H-1), 4.54–4.40 (m, 6 H, 6 × OH-6), 3.83–3.72 (m, 2 H, H-5^I, H-6), 3.70–3.51 (m, 26 H, 7 × H-3, 2 × H-4, 6 × H-5, 11 × H-6), 3.43–3.25 (m, 7 × H-2, 5 × H-4 + H_2O), 3.01–2.95 (m, 1 H, H-6^I), 2.72–2.66 (m, 1 H, H-6^I), 2.75–2.58 (m, 2 H, 2 × H-1′), 2.52–2.51 (m, 2 H, 2 × H-3′), 2.32–2.20 (br s, 1 H, SH), 1.77–1.72 (m, 2 H, 2 × H-2′) ppm. ^{13}C NMR (150 MHz, d_6-DMSO): δ = 102.36 (C-1), 101.98 (5 × C-1), 101.61 (C-1), 85.08 (C-4^I), 81.54–81.42 (5 × C-4), 81.42 (C-4), 73.08–72.04 (7 × C-2, 7 × C-3, 6 × C-5), 71.04 (C-5^I), 59.93–59.85 (6 × C-6), 33.36 (C-2′), 33.14 (C-6^I), 30.76 (C-1′), 22.75 (C-3′) ppm. Content of the non-oxidized product in the NMR spectrum according to the ^{1}H NMR: 91%. IR (drift KBr): υ = 1156, 1078, 1033, 576 cm^{-1}. MS (ESI): $[M+Na]^+$ = 1247.5. For $C_{45}H_{76}NaO_{34}S_2$ calculated 1247.3557.

6^I-Deoxy-6^I-((5-sulfanylpentane-1-yl)sulfanyl)-β-CD (5f)

By the general procedure GP1 181 mg of compound **5f** was obtained from cyclodextrin derivative **1** (298 mg, 0.23 mmol) and pentane-1,5-dithiol in the form of a white solid in 62% yield. TLC – R_f = 0.54. Column chromatography– BuOH/EtOH/H_2O 5/4/4. ^{1}H NMR (600 MHz, d_6-DMSO): δ = 5.83–5.64 (m, 14 H, 7 × OH-2, 7 × OH-3), 4.89–4.76 (m, 7 H, 7 × H-1), 4.50 (t, J = 5.6 Hz, 1 H, OH-6), 4.48–4.42 (m, 4 H, 4 × OH-6), 4.31 (t, J = 5.1 Hz, 1 H, OH-6), 3.84–3.72 (m, 2 H, H-5^I, H-6), 3.72–3.48 (m, 26 H, 7 × H-3, 2 × H-4, 6 × H-5, 11 × H-6), 3.42–3.23 (m, 7 × H-2, 5 × H-4 + H_2O), 3.01–2.93 (m, 1 H, H-6^I), 2.72–2.66 (m, 1 H, H-6^I), 2.55–2.52 (m, 2 H, 2 × H-1′), 2.47–2.42 (m, 2 H, 2 × H-5′), 2.15 (t, J = 7.8 Hz, 1 H, SH), 1.56–1.44 (m, 4 H, 2 × H-2′, 2 × H-4′), 1.43–1.34 (m, 2 H, 2 × H-3′) ppm. ^{13}C NMR (150 MHz, d_6-DMSO): δ = 102.29 (C-1), 101.94 (5 × C-1), 101.58 (C-1), 85.00 (C-4^I), 81.67–81.27 (6 × C-4), 73.04–72.03 (7 × C-2, 7 × C-3, 6 × C-5),

71.13 (C-5[I]), 60.35–59.88 (6 × C-6), 32.91 (C-6[I]), 32.20 (C-1´), 28.67 (C-2´, C-4´), 26.84 (C-3´), 23.64 (C-5´) ppm. Content of the non-oxidized product in the NMR spectrum according to the ^{1}H NMR: 84%. IR (drift KBr): υ = 1156, 1075, 1033, 585 cm^{-1}. MS (ESI): $[M+Na]^+$ = 1275.4. For $C_{47}H_{80}NaO_{34}S_2$ calculated 1275.3870.

6[I]-Deoxy-6[I]-((8-sulfanyloktane-1-yl)sulfanyl)-β-CD (5g)

By the general procedure GP1 210 mg of compound **5g** was obtained from cyclodextrin derivative **1** (303 mg, 0.24 mmol) and octane-1,8-dithiol in the form of a white solid in 69% yield. TLC – R_f = 0.63. Column chromatography – BuOH/EtOH/H_2O 5/4/4. ^{1}H NMR (600 MHz, d_6-DMSO): δ = 5.86–5.52 (m, 14 H, 7 × OH-2, 7 × OH-3), 4.86 (d, *J* = 3.5 Hz, 1 H, H-1), 4.85–4.80 (m, 6 H, 6 × H-1), 4.55–4.32 (m, 6 H, 6 × OH-6), 3.79–3.73 (m, 2 H, H-5[I], H-6), 3.69–3.51 (m, 26 H, 7 × H-3, 2 × H-4, 6 × H-5, 11 × H-6), 3.41–3.27 (m, 7 × H-2, 5 × H-4 + H_2O), 2.99–2.94 (m, 1 H, H-6[I]), 2.74–2.70 (m, 1 H, H-6[I]), 2.52 (t, *J* = 1.9 Hz, 2 H, 2 × H-1´), 2.48–2.45 (m, 2 H, 2 × H-8´), 2.13 (t, *J* =7.7 Hz, 1 H, SH), 1.56–1.51 (m, 2 H, 2 × H-7´), 1.51–1.44 (m, 2 H, 2 × H-2´), 1.38–1.20 (m, 8 H, 2 × H-3´, 2 × H-4´, 2 × H-5´, 2 × H-6´) ppm. ^{13}C NMR (150 MHz, d_6-DMSO): δ = 102.30 (C-1), 101.95 (5 × C-1), 101.67 (C-1), 84.84 (C-4[I]), 81.53–81.40 (6 × C-4), 73.05–72.03 (7 × C-2, 7 × C-3, 6 × C-5), 71.26 (C-5[I]), 59.90 (6 × C-6), 33.24 (C-7´), 33.11 (C-6[I]), 32.44 (C-1´), 29.24 (C-2´), 28.58–27.77 (C-3´,C-4´, C-5´, C-6´), 23.72 (C-8´) ppm. Content of the non-oxidized product in the NMR spectrum according to the ^{1}H NMR: 46%. IR (drift KBr): υ = 3315, 1156, 1078, 1030 cm^{-1}. MS (ESI): $[M+Na]^+$ = 1317.3. For $C_{50}H_{86}NaO_{34}S_2$ calculated 1317.4340.

Procedure for regeneration of unreacted dithiol from the reaction mixture

The chloroform layer obtained by the extraction of the reaction mixture (see GP1) was evaporated under vacuum. The oily residue was purified by column chromatography on silica gel (50 × weight of oil, mobile phase hexane/EtOAc 4:1). Thanks to this procedure 57–76 % of added dithiol was regenerated.

General procedure for oxidation of cyclodextrin thiols into corresponding disulfides (GP2)

Cyclodextrin thiols **5a–g** (0.025 mmol) were dissolved in a mixture of methanol (3.5 mL), water (3.5 mL) and a conc. solution of ammonia (7 mL). Air was bubbled through the solution by using an aquarium compressor. The reaction process was monitored by TLC (BuOH/EtOH/H_2O 5/4/4, detection by H_2SO_4). Solvents were refilled when needed during the next 3 days. Then all solvents were removed by evaporation under reduced pressure.

Disulfide 6a

By the general procedure GP2 31 mg of compound **6a** was obtained from cyclodextrin derivative **5a** (31 mg, 0.03 mmol) in the form of a white solid in 100% yield. TLC – R_f = 0.26. ^{1}H NMR (600 MHz, d$_6$-DMSO): δ = 5.87–5.63 (m, 28 H, 14 × OH-2, 14 × OH-3), 4.90–4.78 (m, 14 H, 14 × H-1), 4.51–4.31 (m, 12 H, 12 × OH-6), 3.83–3.71 (m, 4 H, 2 × H-5^{I}, 2 × H-6), 3.72–3.47 (m, 52 H, 14 × H-3, 4 × H-4, 12 × H-5, 22 × H-6), 3.47–3.18 (m, 14 × H-2, 10 × H-4, 4 × H-2´, 4 × H-3´ + H_2O), 3.05–2.98 (m, 2 H, 2 × H-6^{I}), 2.88 (t, J = 6.1 Hz, 4 H, 4 × H-4´), 2.80 (dd, J = 13.3, 6.2 Hz, 2 H, 2 × H-6^{I}), 2.77–2.67 (m, 4 H, 4 × H-1´), ppm. ^{13}C NMR (150 MHz, d$_6$-DMSO): δ = 102.28 (2 × C-1), 101.96 (10 × C-1), 101.72 (2 × C-1), 84.69 (2 × C-4^{I}), 81.57–81.51 (12 × C-4), 73.05–72.03 (14 × C-2, 14 × C-3, 12 × C-5), 71.41 (2 × C-5^{I}), 69.78 (2 × C-2´), 68.27 (2 × C-3´), 59.92 (12 × C-6), 37.74 (2 × C-4´), 33.27 (2 × C-6^{I}), 31.87 (2 × C-1´) ppm. IR (drift KBr): υ = 1156, 1078, 1033, 585 cm^{-1}. MS (ESI): [M+Na]$^{+}$ = 2531.1. For $C_{92}H_{154}NaO_{70}S_4$ calculated 2530.73.

Disulfide 6b

By the general procedure GP2 19 mg of compound **6b** was obtained from cyclodextrin derivative **5b** (20 mg, 0.02 mmol) in the form of a white solid in 100% yield. TLC – R_f = 0.23. ^{1}H NMR (600 MHz, d$_6$-DMSO): δ = 5.85–5.63 (m, 28 H, 14 × OH-2, 14 × OH-3), 4.86 (d, J = 3.6 Hz, 2 H, 2 × H-1), 4.85–4.81 (m, 12 H, 12 × H-1), 4.49–4.41 (m, 12 H, 12 × OH-6), 3.80–3.72 (m, 4 H, 2 × H-5^{I}, 2 × H-6), 3.70–3.49 (m, 68 H, 14 × H-3, 4 × H-4, 12 × H-5, 22 × H-6, 4 × H-2´, 4 × H-3´, 4 × H-4´, 4 × H-5´), 3.40–3.26 (m, 14 × H-2, 10 × H-4 + H_2O), 3.03–2.99 (m, 2 H, 2 × H-6^{I}), 2.89 (t, J = 6.4, 4 H, 4 × H-6´), 2.82–2.75 (m, 2 H, 2 × H-6^{I}), 2.74–2.66 (m, 4 H, 4 × H-1´) ppm. ^{13}C NMR (150 MHz, d$_6$-DMSO): δ = 102.28 (2 × C-1), 101.96 (10 × C-1), 101.70 (2 × C-1), 84.72 (2 × C-4^{I}), 81.56–81.44 (12 × C-4), 73.05–72.03 (14 × C-2, 14 × C-3, 12 × C-5), 71.41 (2 × C-5^{I}), 70.01 (2 × C-2´), 69.54–69.37 (2 × C-3´, 2 × C-4´), 68.64 (2 × C-5´), 59.92 (12 × C-6), 37.75 (2 × C-6´), 33.27 (2 × C-6^{I}), 31.86 (2 × C-1´) ppm. IR (drift KBr): υ = 1153, 1078, 1027, 576 cm^{-1}. MS (ESI): [M+Na]$^{+}$ = 2619.1. For $C_{96}H_{162}NaO_{72}S_4$ calculated 2568.7931.

Disulfide 6c

By the general procedure GP2 43 mg of compound **6c** was obtained from cyclodextrin derivative **5c** (45 mg, 0,03 mmol) in the form of a white solid in 96% yield. TLC – R_f = 0.21. ^{1}H NMR (600 MHz, d$_6$-DMSO): δ = 5.80–5.66 (m, 28 H, 14 × OH-2, 14 × OH-3), 4.85 (d, J = 3.5 Hz, 2 H, 2 × H-1), 4.84–4.80 (m, 12 H, 12 × H-1), 4.50–4.43 (m, 10 H, 10 × OH-6), 4.34 (t, J = 5.2 Hz, 2 H, 2 × OH-6), 3.79–3.71 (m, 4 H, 2 × H-5^{I}, 2 × H-6), 3.70–3.47 (m, 76 H, 14 × H-3, 4 × H-4, 12 × H-5, 22 × H-6, 4 × H-2´, 4 × H-3´, 4 × H-4´, 4 × H-5´, 4 × H-6´, 4 × H-7´), 3.41–3.27 (m, 14 × H-2, 10 × H-4 + H_2O), 3.03–2.98 (m, 2 H, 2 × H-6^{I}), 2.89 (t, J = 6.4 Hz, 4 H, 4 × H-8´), 2.27 (dd, J = 13.9, 6.9 Hz, 2 H, 2 × H-6^{I}), 2.75–2.66 (m, 4 H, 4 ×

H-1′) ppm. ^{13}C NMR (150 MHz, d_6-DMSO): δ = 102.33 (2 × C-1), 102.01 (10 × C-1), 101.74 (2 × C-1), 84.76 (2 × C-4^{I}), 81.60–81.48 (12 × C-4), 73.10–72.04 (14 × C-2, 14 × C-3, 12 × C-5), 71.44 (2 × C-5^{I}), 70.03–69.49 (2 × C-2′, 2 × C-3′, 2 × C-4′, 2 × C-5′, 2 × C-6′), 68.66 (2 × C-7′), 59.97 (12 × C-6), 37.87 (2 × C-8′), 33.32 (2 × C-6^{I}), 31.93 (2 × C-1′) ppm. IR (drift KBr): υ = 1150, 1108, 1057, 471 cm^{-1}. MS (ESI): [M+Na]$^+$ = 2706.9. For $C_{100}H_{170}NaO_{74}S_4$ calculated 2706.84.

Disulfide 6d

By the general procedure GP2 32 mg of compound **6d** was obtained from cyclodextrin derivative **5d** (32 mg, 0,02 mmol) in the form of a white solid in 100% yield. TLC – R_f = 0.30. ^{1}H NMR (600 MHz, d_6-DMSO): δ = 5.81–5.65 (m, 28 H, 14 × OH-2, 14 × OH-3), 4.86 (d, J = 3.4 Hz, 2 H, 2 × H-1), 4.85–4.80 (m, 12 H, 12 × H-1), 4.50–4.42 (m, 12 H, 12 × OH-6), 3.81–3.76 (m, 2 H, 2 × H-5^{I}), 3.76–3.70 (m, 2 H, 2 × H-6), 3.70–3.52 (m, 52 H, 14 × H-3, 4 × H-4, 12 × H-5, 22 × H-6), 3.40–3.27 (m, 14 × H-2, 10 × H-4 + H_2O), 3.05–3.00 (m, 2 H, 2 × H-6^{I}), 2.93–2.88 (m, 4 H, 4 × H-2′), 2.88–2.80 (m, 6 H, 2 × H-6^{I}, 4 × H-1′) ppm. ^{13}C NMR (150 MHz, d_6-DMSO): δ = 102.27 (2 × C-1), 101.96 (10 × C-1), 101.71 (2 × C-1), 84.60 (2 × C-4^{I}), 81.67–81.49 (12 × C-4), 73.06–72.03 (14 × C-2, 14 × C-3, 12 × C-5), 71.31 (2 × C-5^{I}), 60.09–59.85 (12 × C-6), 38.03 (2 × C-2′), 32.96 (2 × C-6^{I}), 31.92 (2 × C-1′) ppm. IR (drift KBr): υ = 3312, 1162, 1078, 1036 cm^{-1}MS (ESI): [M+Na]$^+$ = 2442.1. For $C_{88}H_{146}NaO_{68}S_4$ calculated 2441.67.

Disulfide 6e

By the general procedure GP2 81 mg of compound **6e** was obtained from cyclodextrin derivative 5**e** (82 mg, 0.07 mmol) and in the form of a white solid in 99% yield. TLC – R_f = 0.30. ^{1}H NMR (600 MHz, d_6-DMSO): δ = 5.87–5.62 (m, 28 H, 14 × OH-2, 14 × OH-3), 4.88–4.86 (m, 2 H, 2 × H-1), 4.85–4.79 (m, 12 H, 12 × H-1), 4.50–4.39 (m, 12 H, 12 × OH-6), 3.81–3.72 (m, 4 H, 2 × H-5^{I}, 2 × H-6), 3.70–3.50 (m, 52 H, 14 × H-3, 4 × H-4, 12 × H-5, 22 × H-6), 3.43–3.23 (m, 14 × H-2, 10 × H-4 + H_2O), 3.02–2.96 (m, 2 H, 2 × H-6^{I}), 2.75 (t, J = 6.5 Hz, 4 H, 4 × H-3′), 2.75–2.58 (m, 6 H, 2 × H-6^{I}, 4 × H-1′), 1.85 (t, J = 6.7 Hz, 4 H, 4 × H-2′) ppm. ^{13}C NMR (150 MHz, d_6-DMSO): δ = 102.30 (2 × C-1), 101.96 (10 × C-1), 101.71 (2 × C-1), 84.75 (2 × C-4^{I}), 81.53–81.48 (12 × C-4), 73.05–72.04 (14 × C-2, 14 × C-3, 12 × C-5), 71.26 (2 × C-5^{I}), 59.98–59.94 (12 × C-6), 36.26 (2 × C-3′), 33.03 (2 × C-6^{I}), 30.94 (2 × C-1′), 28.52 (2 × C-2′) ppm. IR (drift KBr): υ = 1156, 1075, 1030, 626 cm^{-1}. MS (ESI): [M+Na]$^+$ = 2470.2. For $C_{90}H_{150}NaO_{68}S_4$ calculated 2469.71.

Disulfide 6f

By the general procedure GP2 24 mg of compound **6f** was obtained from cyclodextrin derivative **5f** (25 mg, 0,02 mmol) and in the form of a white solid in 96% yield. TLC – R_f = 0.33. ^{1}H NMR (600 MHz, d_6-DMSO): δ = 5.80–5.64 (m, 28 H, 7 × OH-2, 7 × OH-3), 4.86 (d, J = 3.4 Hz, 2 H, 2 × H-1), 4.84–4.80 (m, 12 H, 12 × H-1), 4.50–4.41 (m, 12 H, 12 ×

OH-6), 3.81–3.73 (m, 4 H, 2 × H-5^{I}, 2 × H-6), 3.72–3.48 (m, 52 H, 14 × H-3, 4 × H-4, 12 × H-5, 22 × H-6), 3.45–3.24 (m, 14 × H-2, 10 × H-4 + H_2O), 3.01–2.93 (m, 2 H, 2 × H-6^{I}), 2.76–2.70 (m, 2 H, 2 × H-6^{I}), 2.68 (t, J = 7.2 Hz, 4 H, 4 × H-5´), 2.56–2.53 (m, 4 H, 4 × H-1´), 1.64–1.57 (m, 4 H, 4 × H-2´) 1.54–1.46 (m, 4 H, 4 × H-4´), 1.44–1.36 (m, 4 H, 4 × H-3´) ppm. ^{13}C NMR (150 MHz, d_6-DMSO): δ = 102.30 (2 × C-1), 101.96 (10 × C-1), 101.71 (2 × C-1), 84.81 (2 × C-4^{I}), 81.52–81.46 (12 × C-4), 73.05–72.04 (14 × C-2, 14 × C-3, 12 × C-5), 71.30 (2 × C-5^{I}), 59.91 (12 × C-6), 37.62 (2 × C-5´), 33.06 (2 × C-6^{I}), 32.34 (2 × C-1´), 28.82 (2 × C-2´), 28.21(2 × C-4´), 27.02 (2 × C-3´) ppm. IR (drift KBr): υ = 1153, 1075, 1033, 579 cm^{-1}. MS (ESI): [M+Na]$^{+}$ = 2527.1. For $C_{94}H_{158}NaO_{68}S_4$ calculated 2526.77.

Disulfide 6g

By the general procedure GP2 31 mg of compound **6g** was obtained from cyclodextrin derivative **5g** (32 mg, 0,02 mmol) in the form of a white solid in 98% yield. TLC – R_f = 0.35. ^{1}H NMR (600 MHz, d_6-DMSO): δ = 5.93–5.44 (m, 28 H, 14 × OH-2, 14 × OH-3), 4.86 (d, J = 3.4 Hz, 2 H, 2 × H-1), 4.85–4.79 (m, 12 H, 12 × H-1), 4.64–4.20 (m, 12 H, 12 × OH-6), 3.79–3.73 (m, 4 H, 2 × H-5^{I}, 2 × H-6), 3.71–3.49 (m, 52 H, 14 × H-3, 4 × H-4, 12 × H-5, 22 × H-6), 3.46–3.21 (m, 14 × H-2, 10 × H-4 + H_2O), 2.99–2.93 (m, 2 H, 2 × H-6^{I}), 2.75–2.70 (m, 2 H, 2 × H-6^{I}), 2.68 (t, J = 7.2 Hz, 4 H, 4 × H-8´), 2.55–2.51 (m, 4 H, 4 × H-1´), 1.63–1.57 (m, 4 H, 4 × H-7´), 1.51–1.44 (m, 4 H, 4 × H-2´), 1.38–1.19 (m, 16 H, 4 × H-3´, 4 × H-4´, 4 × H-5´, 4 × H-6´) ppm. ^{13}C NMR (150 MHz, d_6-DMSO): δ = 102.30 (2 × C-1), 101.95 (10 × C-1), 101.68 (2 × C-1), 84.85 (2 × C-4^{I}), 81.55–81.41 (12 × C-4), 73.05–72.04 (14 × C-2, 14 × C-3, 12 × C-5), 71.27 (2 × C-5^{I}), 59.91 (12 × C-6), 37.75 (2 × C-8´) 33.12 (2 × C-6^{I}), 32.45 (2 × C-1´), 29.25 (2 × C-2´), 28.59 (2 × C-7´), 28.56–27.77 (2 × C-3´,2 × C-4´, 2 × C-5´, 2 × C-6´) ppm. IR (drift KBr): υ = 1144, 1033, 641, 620 cm^{-1}. MS (ESI): [M+Na]$^{+}$ = 2611.3. For $C_{100}H_{170}O_{68}S_4$ calculated 2610.87.

Procedure for reduction of cyclodextrin disulfide to the corresponding thiol

Cyclodextrin disulfide **6e** (30 mg, 0.01 mmol) was dissolved under inert atmosphere in DMF (1 mL) and a 2 M solution of NaOH (0.25 ml). Ethanethiol (1 mL, 13.2 mmol) was added and the reaction mixture was stirred at room temperature for 12 hours. The reaction process was monitored by TLC (BuOH/EtOH/H_2O 5/4/4, detection by H_2SO_4). Then the solution was diluted by addition of 50% aqueous MeOH (0.5 mL) and poured to acetone (30 mL). The white precipitate was separated by centrifugation (6000 RPM, 6 min), dissolved in 50% aqueous MeOH and poured into acetone again. This procedure was repeated once again. After last separation, the precipitate was dried under vacuum. This procedure gave 24 mg of cyclodextrin thiol **5e** in 80% yield.

General procedure of ditosylation of oligo ethylene glycols (GP3)

Compounds **2a–c** were prepared by a modified procedure known from the literature[1]. Oligo ethylene glycol (37.7 mmol) and *p*-toluenesulfonyl chloride (14.4 g, 75.4 mmol, 2 equiv) were dissolved in dichloromethane (36 mL) and this solution was cooled to 0 °C. During permanent stirring, milled potassium hydroxide (17 g, 302 mmol, 8 equiv) was added and the solution was then stirred for 3 hours while the temperature was kept from 0 to 10 °C. This reaction was monitored by TLC (UV detection). After warming up to room temperature the reaction mixture was diluted by chloroform and extracted with water (3 × 1:1). Chloroform extracts were collected, dried over magnesium sulfate overnight, filtered and solvents were evaporated under reduced pressure.

3-Oxapentan-1,5-diyl-bis(*p*-toluensulfonate) (2a)

By the general procedure GP3 15.408 g of compound **2a** was obtained from diethylene glycol (4.000 g, 37.7 mmol) in the form of a white solid in 99% yield. TLC – hexane/EtOAc 2/1, R_f = 0.20. ^{1}H NMR (300 MHz, $CDCl_3$): δ = 7.76 (d, *J* = 8.2 Hz, 4 H, 4 × CH), 7.32 (d, *J* = 8.1 Hz, 4 H, 4 × CH), 4.07 (t, *J* = 4.7 Hz, 4 H, 2 × CH_2), 3.59 (t, *J* = 4.7 Hz, 4 H, 2 × CH_2), 2.43 (s, 6 H, 2 × CH_3) ppm, in agreement with the literature[1].

3,6-Dioxaoctane-1,8-diyl-bis(*p*-toluensulfonate) (2b)

By the general procedure GP3 30.639 g of compound **2b** was obtained from triethylene glycol (10.113 g, 67.3 mmol) in the form of a white gel substance in 99% yield. TLC – hexane/EtOAc 1/1, R_f = 0.43. ^{1}H NMR (300 MHz, $CDCl_3$): δ = 7.77 (d, *J* = 8.4 Hz, 4 H, 4 × CH), 7.32 (d, *J* = 8.0 Hz, 4 H, 4 × CH), 4.12 (t, *J* = 4.7 Hz, 4 H, 2 × CH_2), 3.63 (t, *J* = 4.8 Hz, 4 H, 2 × CH_2), 3.50 (s, 4 H, 2 × CH_2), 2.42 (s, 6 H, 2 × CH_3) ppm, in agreement with the literature[1].

3,6,9-Trioxaundecane-1,11-diyl-bis(*p*-toluensulfonate) (2c)

By the general procedure GP3 25.494 g of compound **2c** was obtained from tetraethylene glycol (10.008 g, 51.53 mmol) in the form of a white gel substance in 98% yield. TLC – hexane/EtOAc 1/1, R_f = 0.25. ^{1}H NMR (300 MHz, $CDCl_3$): δ = 7.76 (d, *J* = 8.3 Hz, 4 H, 4 × CH), 7.31 (d, *J* = 8.6 Hz, 4 H, 4 × CH), 4.13 (t, *J* = 4.8 Hz, 4 H, 2 × CH_2), 3.68–3.51 (m, 12 H, 6 × CH_2), 2.42 (s, 6 H, 2 × CH_3) ppm, in agreement with the literature[1].

General procedure for bis(thioacetylation) ditosylated oligo ethylene glycols (GP4)

Compounds 3**a – 3c** were prepared by a modified procedure known from the literature[2]. Ditosylated oligo ethylene glycol **2a**, **2b** or **2c** (3 mmol) was suspended with potassium thioacetate (1.7 g, 15 mmol, 5 equiv) and sodium iodide (45 mg, 0.3 mmol, 0.1 equiv) in acetone (80 mL). The reaction mixture was then stirred under a reflux

condenser at 60 °C for 18 hours. During this time the reaction mixture changed color from white to yellow-orange. The reaction process was monitored by TLC (detection by $KMnO_4$). After cooling to room temperature, a minimal amount of water was added for solubilization of solid particles. The solution was then extracted with chloroform (1:1) and the organic phase was washed with water (2 × 1:1) and concentrated under reduced pressure. The residue was purified by column chromatography on silica gel (100 × weight of the residue).

S,S'-(3-Oxapentane-1,5-diyl)-bis(thioacetate) (3a)

By the general procedure GP4 647 mg of compound **3a** was obtained from **2a** (1.500 g, 3.62 mmol) in the form of an orange oil in 80% yield. TLC – hexane/EtOAc 3/1, R_f = 0.52. Column chromatography – hexane/EtOAc 4/1. ^{1}H NMR (300 MHz, $CDCl_3$): δ = 3.56 (t, J = 6.4 Hz, 4 H, 2 × CH_2), 3.05 (t, J = 6.4 Hz, 4 H, 2 × CH_2), 2.32 (s, 6 H, 2 × CH_3) ppm, in agreement with the literature[2].

S,S'-(3,6-Dioxaoctane-1,8-diyl)-bis(thioacetate) (3b)

By the general procedure GP4 49 mg of compound **3b** was obtained from **2b** (100 mg, 0.23 mmol) in the form of a dark orange oil in 86% yield. TLC – hexane/EtOAc 3/1, R_f = 0.39. Column chromatography – hexane/EtOAc 4/1. ^{1}H NMR (300 MHz, $CDCl_3$): δ = 3.62–3.55 (m, 8 H, 4 × CH_2), 3.08 (t, J = 6.4 Hz, 4 H, 2 × CH_2), 2.32 (s, 6 H, 2 × CH_3) ppm, in agreement with the literature[2].

S,S'-(3,6,9-Trioxaundecane-1,11-diyl)-bis(thioacetate) (3c)

By the general procedure GP4 749 mg of compound **3c** was obtained from **2c** (1.500 g, 2.98 mmol) in the form of yellow-orange oil in 94% yield. TLC – hexane/EtOAc 1/2, R_f = 0.65. Column chromatography – $CHCl_3$/MeOH 70/1. ^{1}H NMR (300 MHz, $CDCl_3$): δ = 3.63–3.55 (m, 12 H, 6 × CH_2), 3.08 (t, J = 6.5 Hz, 4 H, 2 × CH_2), 2.32 (s, 6 H, 2 × CH_3) ppm, in agreement with the literature[3].

General procedure for deacetylation of bis(thioacetylated) oligo ethylene glycols (GP5)

Compounds 4**a–c** were prepared by a modified procedure known from the literature[3]. Bis(thioacetylated) oligo ethylene glycol **3a**, **3b** or **3c** (3.1 mmol) was dissolved in a mixture of methanol (5 mL), water (5 mL) and hydrochloric acid (0.8 mL, 9 mmol, 3 equiv). Argon was bubbled through the mixture of methanol, water and acid in advance (2 minutes), exposed to ultrasound (10 minutes) and argon was bubbled through by again (10 minutes) to remove all dissolved oxygen. The reaction mixture was then heated to 120 °C under a reflux condenser and inert atmosphere for 3 hours. The reaction process was monitored by TLC (detection by $KMnO_4$). After cooling to room temperature, the reaction mixture was diluted to the double volume by addition of water and extracted with chloroform (3 × 1:1).

The organic layers were collected and concentrated under reduced pressure. The residue was purified by column chromatography on silica gel (150 × weight of the residue).

3-Oxapentane-1,5-dithiol (4a)

By the general procedure GP5 266 mg of compound **4a** was obtained from **3a** (580 mg, 2.61 mmol) in the form of a yellow oil in 74% yield. TLC – hexane/EtOAc 3/1, R_f = 0.47. Column chromatography – hexane/EtOAc 4/1. ^{1}H NMR (300 MHz, $CDCl_3$): δ = 3.59 (t, J = 6.3 Hz, 4 H, 2 × CH_2), 2.68 (dt, J = 8.2, 6.3 Hz, 4 H, 2 × CH_2), 1.58 (t, J = 8.2 Hz, 2 H, 2 × SH) ppm, in agreement with the literature[4].

3,6-Dioxaoctane-1,8-dithiol (4b)

By the general procedure GP5 411 mg of compound **4b** was obtained from **3b** (779 mg, 3.09 mmol) in the form of a light-yellow oil in 73% yield. TLC – hexane/EtOAc 3/1, R_f = 0.35. Column chromatography – hexane/EtOAc 4/1. ^{1}H NMR (300 MHz, $CDCl_3$): δ = 3.63–3.58 (m, 8 H, 4 × CH_2), 2.68 (dt, J = 8.2, 6.4 Hz, 4 H, 2 × CH_2), 1.57 (t, J = 8.2 Hz, 2 H, 2 × SH) ppm, in agreement with the literature[2].

3,6,9-Trioxaundecane-1,11-dithiol (4c)

By the general procedure GP5 423 mg of compound **4c** was obtained from **3c** (675 mg, 2.53 mmol) in the form of a light-yellow oil in 74% yield. TLC – hexane/EtOAc 1/2, R_f = 0.59. Column chromatography – hexane/EtOAc 3/1. ^{1}H NMR (300 MHz, $CDCl_3$): δ = 3.68–3.57 (m, 12 H, 6 × CH_2), 2.68 (dt, J = 8.1, 6.4 Hz, 4 H, 2 × CH_2), 1.57 (t, J = 8.1 Hz, 2 H, 2 × SH) ppm, in agreement with the literature[3].

References

1. Bonger, K. M.; van den Berg, R. J. B. H. N.; Heitman, L. H.; IJzerman, A. P.; Oosterom, J.; Timmers, C. M.; Overkleeft, H. S.; van der Marel, G. A. *Bioorgan. Med. Chem.* **2007**, *15*, 4841–4856.
2. Scullion, L. E.; Leary, E.; Higgins, S. J.; Nichols, R. J. *J. Phys.-Condens. Mat.* **2012**, *24*, 164211–164219.
3. Keddie, D. J.; Grande, J. B.; Gonzaga, F.; Brook, M. A.; Dargaville, T. R. *Org. Lett.* **2011**, *13*, 6006–6009.
4. Roy, R. K.; Ramakrishnan, S. *J. Polym. Sci. Pol. Chem.* **2013**, *51*, 4125–4135.
5. Nelles, G.; Weisser, M.; Back, R.; Wohlfart, P.; Wenz, G.; Mittler-Neher, S. *J. Am. Chem. Soc.* **1996**, *118*, 5039–5046.

^{1}H and ^{13}C NMR spectra

6^{I}-Deoxy-6^{I}-((5-sulfanyl-3-oxapentane-1-yl)sulfanyl)-β-CD (5a)

^{1}H

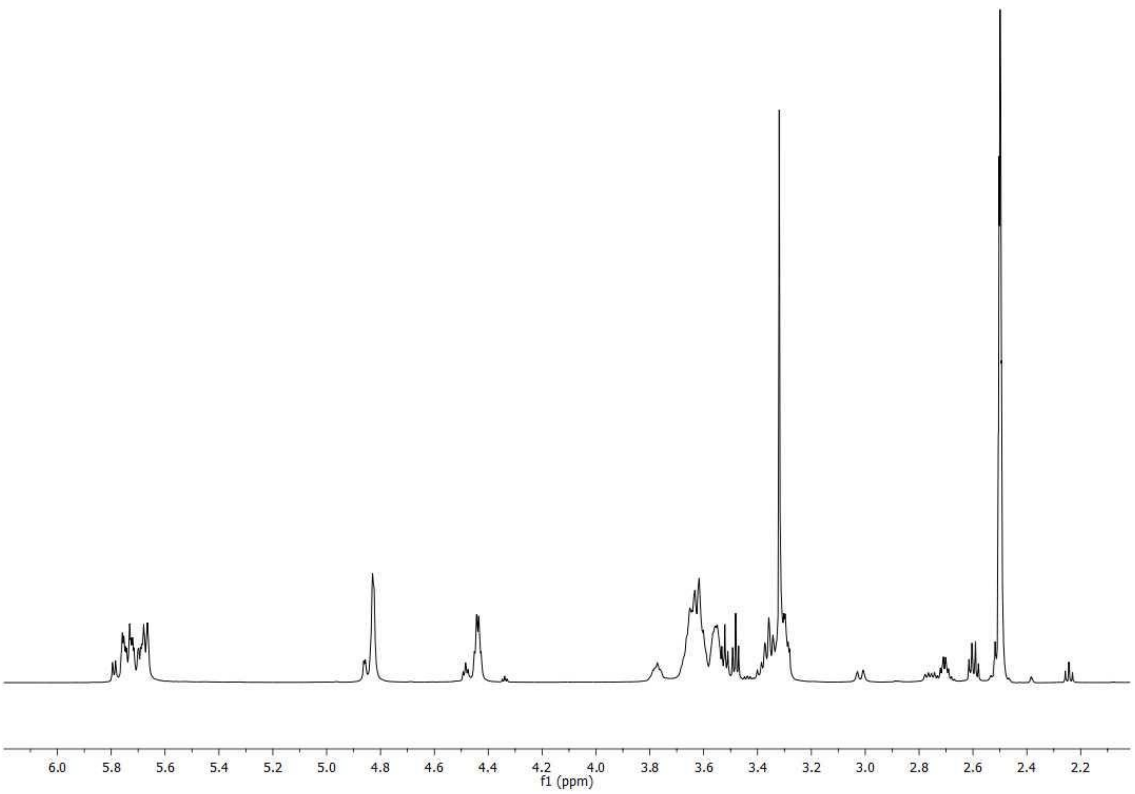

^{13}C

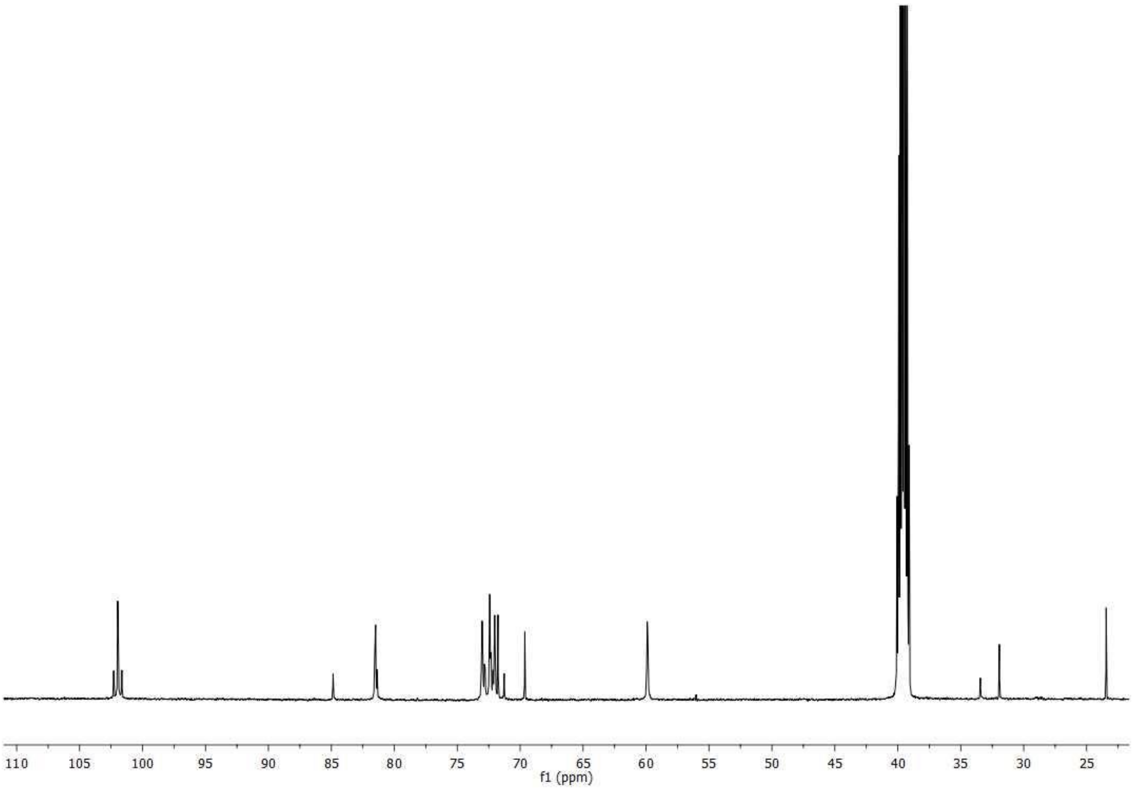

6^I-Deoxy-6^I-((8-sulfanyl-3,6-dioxaoctane-1-yl)sulfanyl)-β-CD (5b)

^{1}H

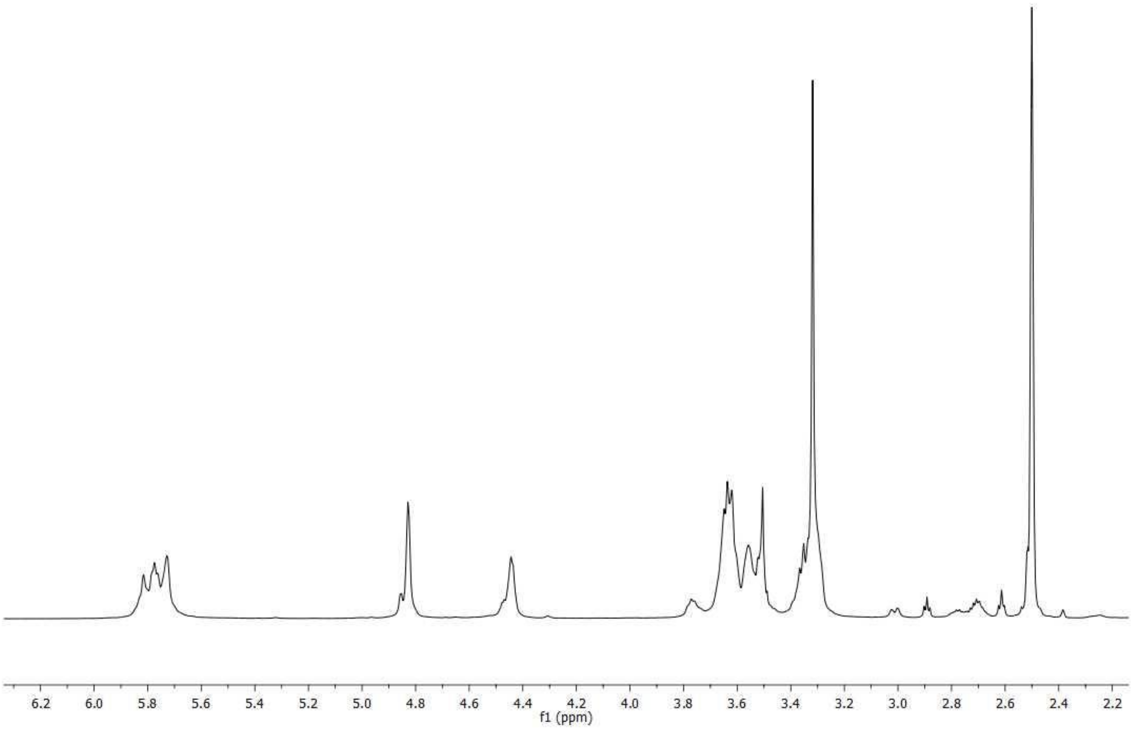

^{13}C

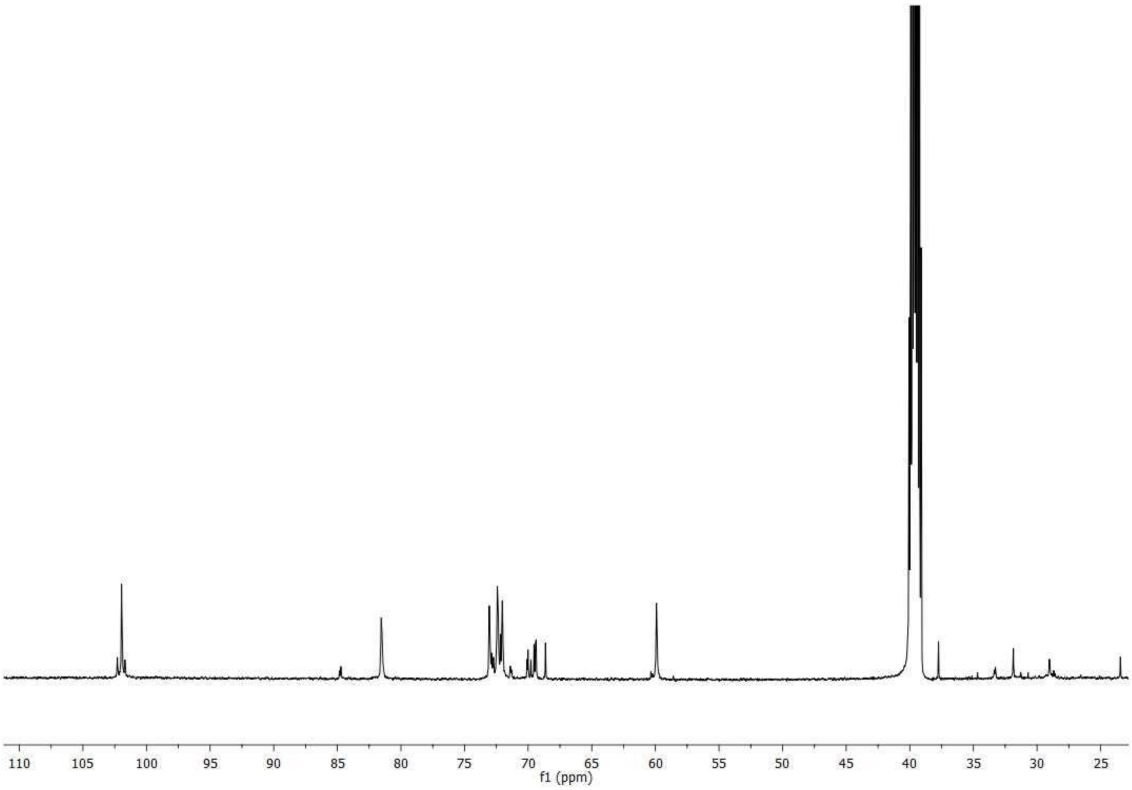

6^I-Deoxy-6^I-((11-sulfanyl-3,6,9-trioxaundecane-1-yl)sulfanyl)-β-CD (5c)

^{1}H

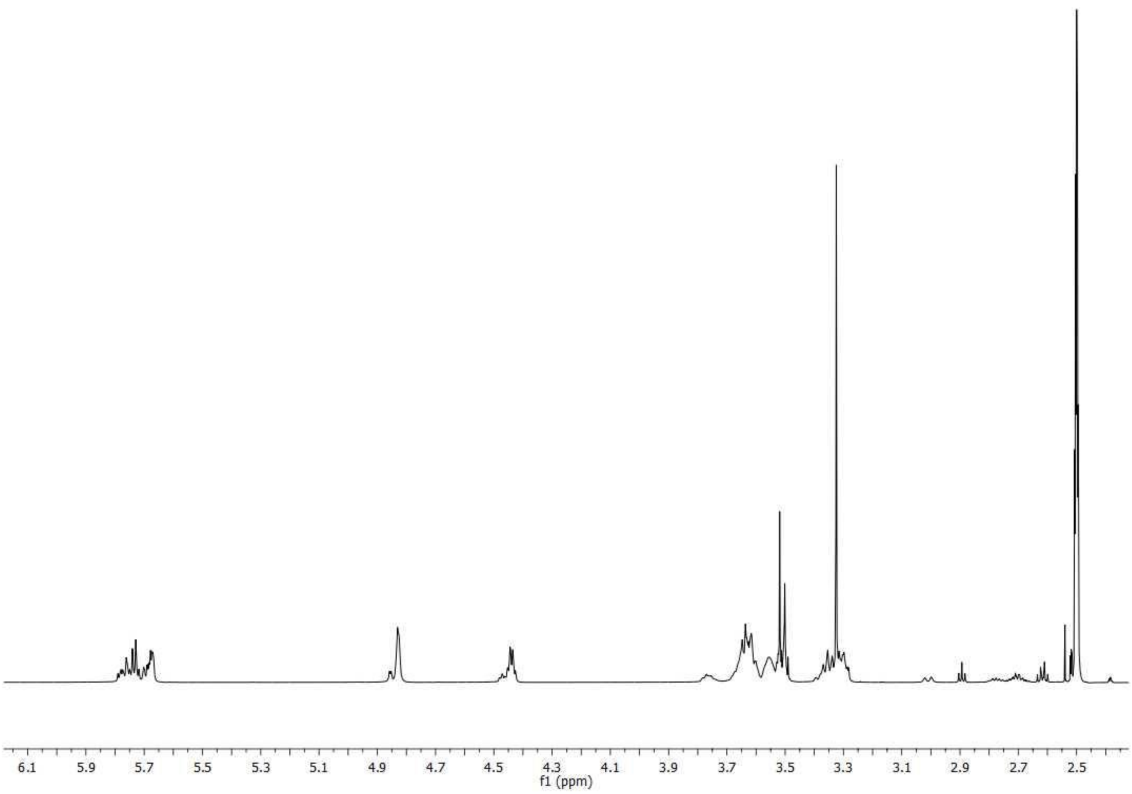

^{13}C

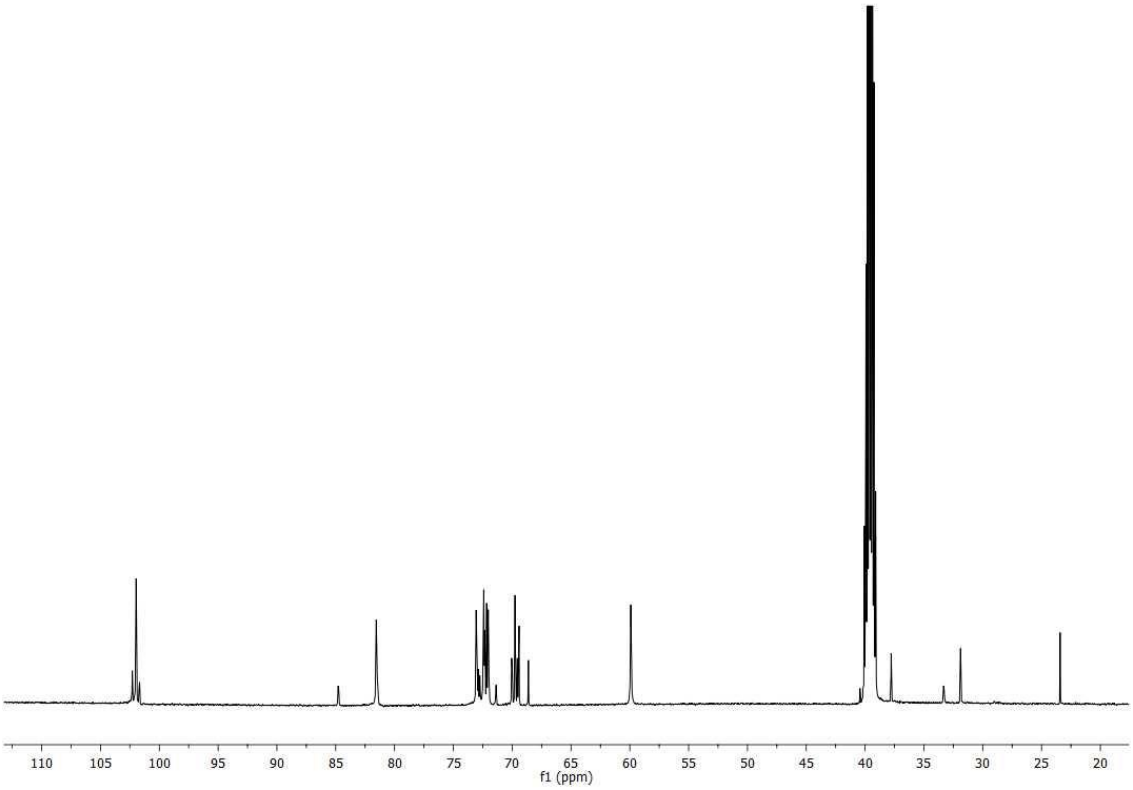

6^I^-Deoxy-6^I^-((2-sulfanylethane-1-yl)sulfanyl)-β-CD (5d)

^{1}H

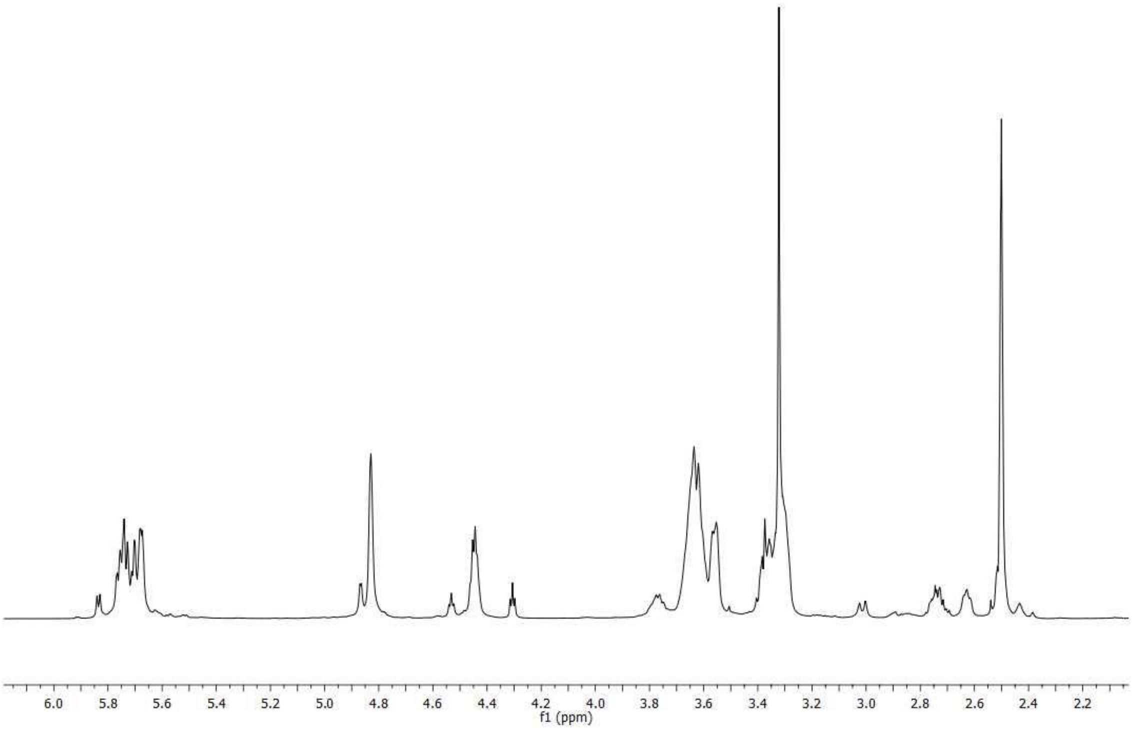

^{13}C

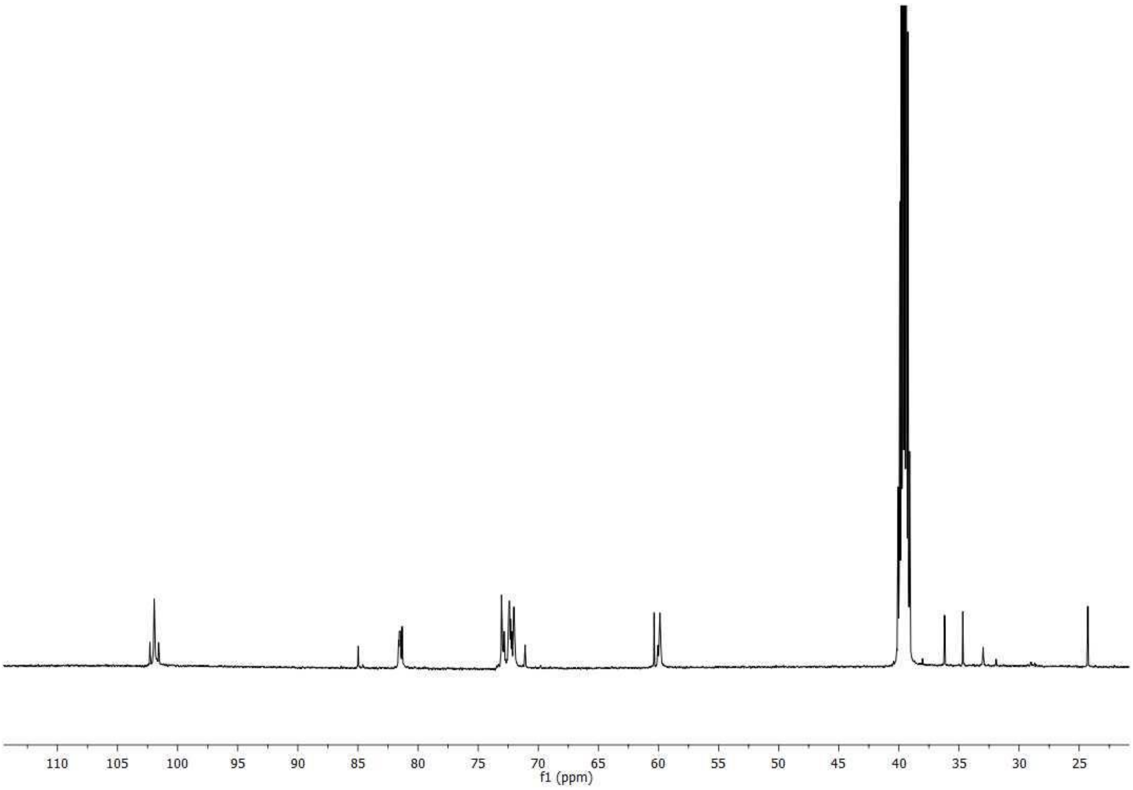

6^I-Deoxy-6^I-((3-sulfanylpropane-1-yl)sulfanyl)-β-CD (5e)

^{1}H

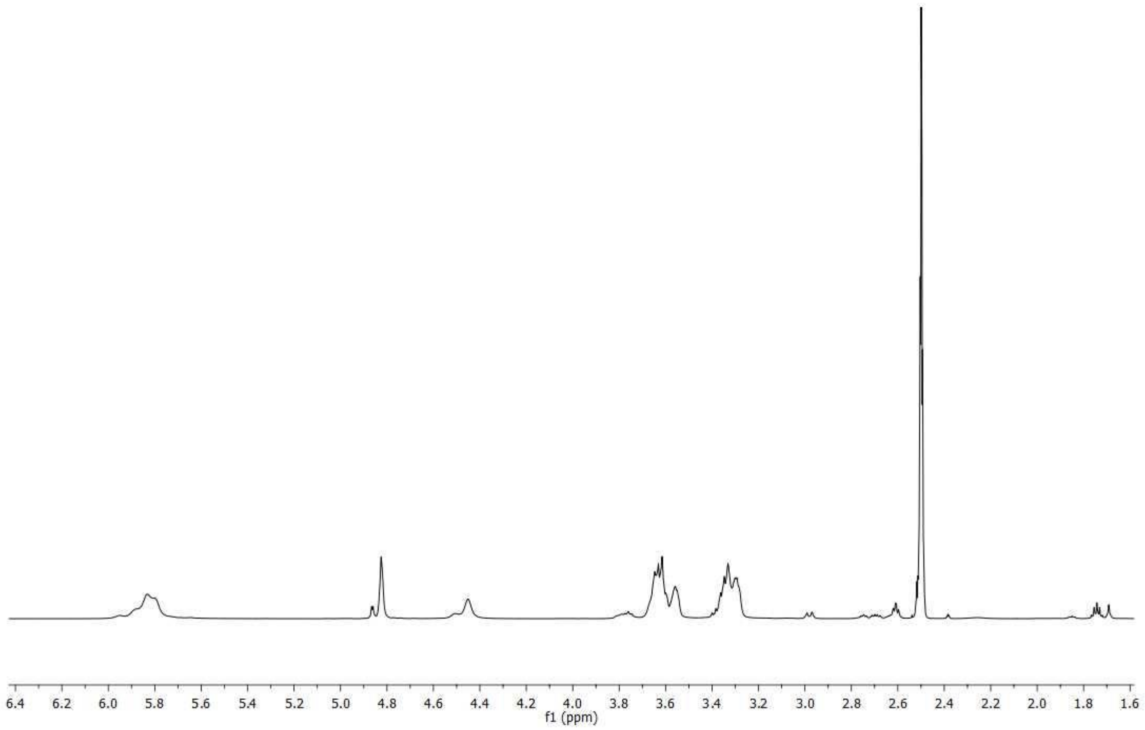

^{13}C

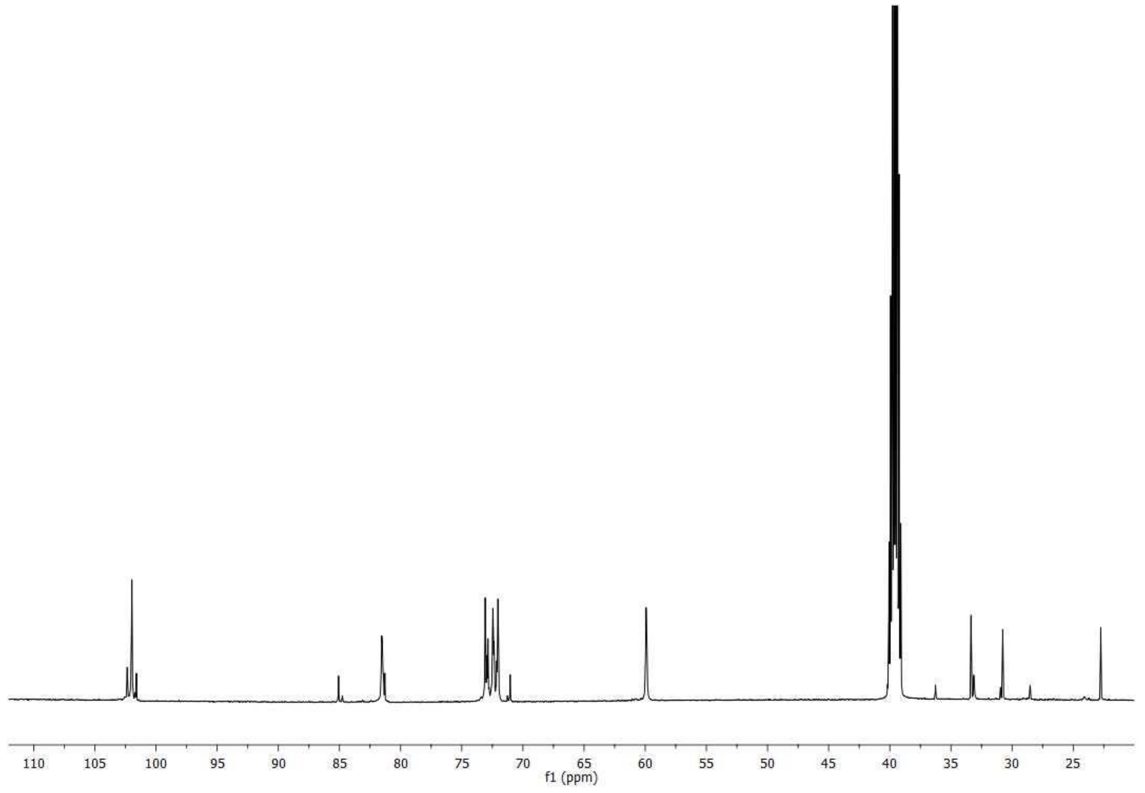

6^I-Deoxy-6^I-((5-sulfanylpentane-1-yl)sulfanyl)-β-CD (5f)

^{1}H

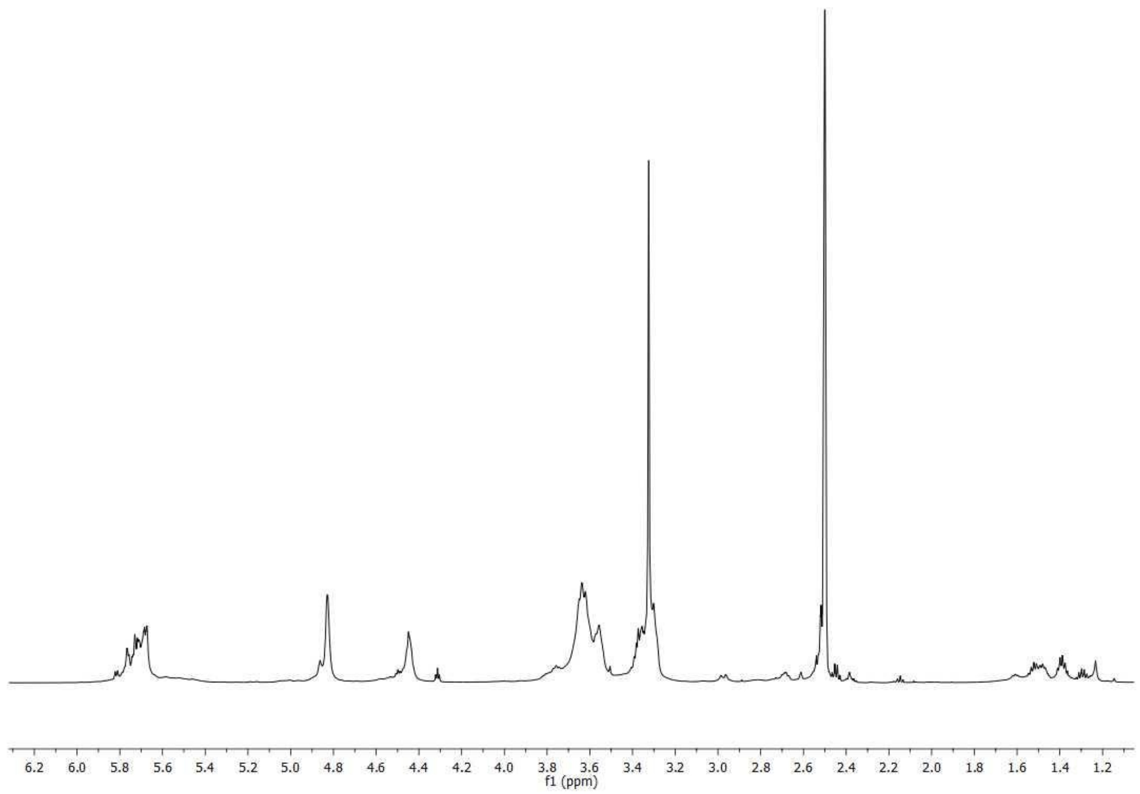

^{13}C

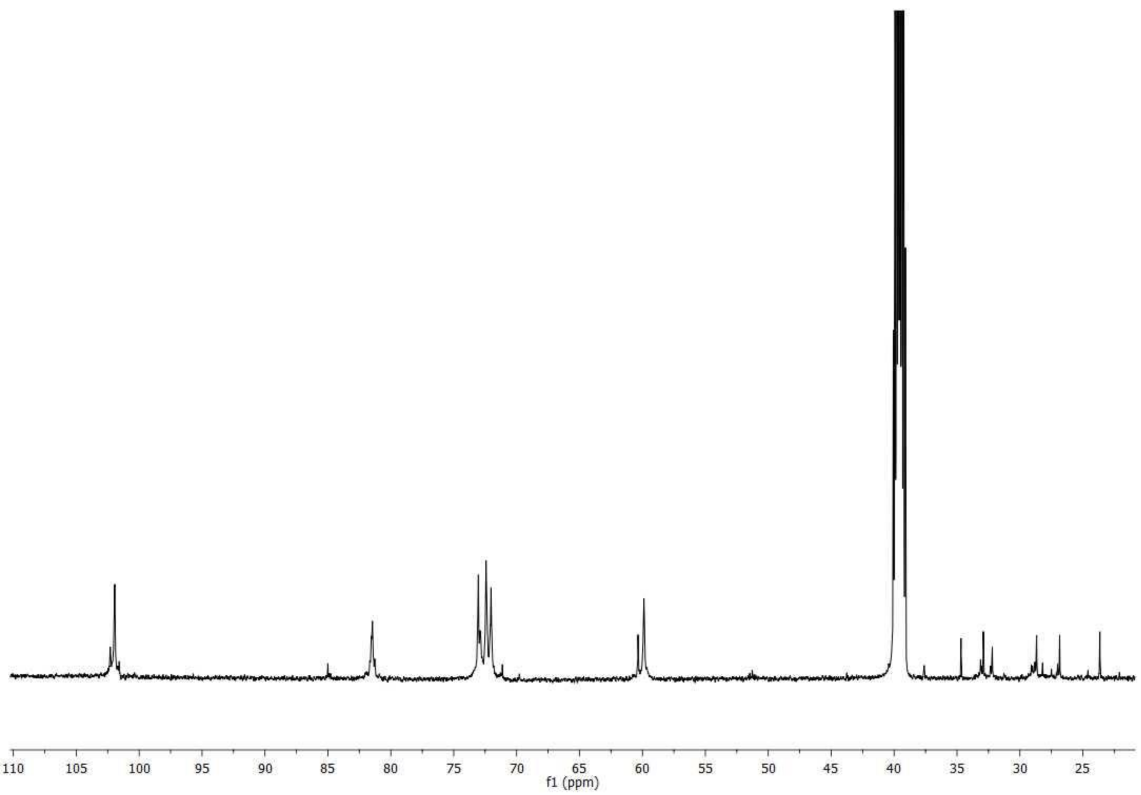

6^I-Deoxy-6^I-((8-sulfanyloktane-1-yl)sulfanyl)-β-CD (5g)

^{1}H

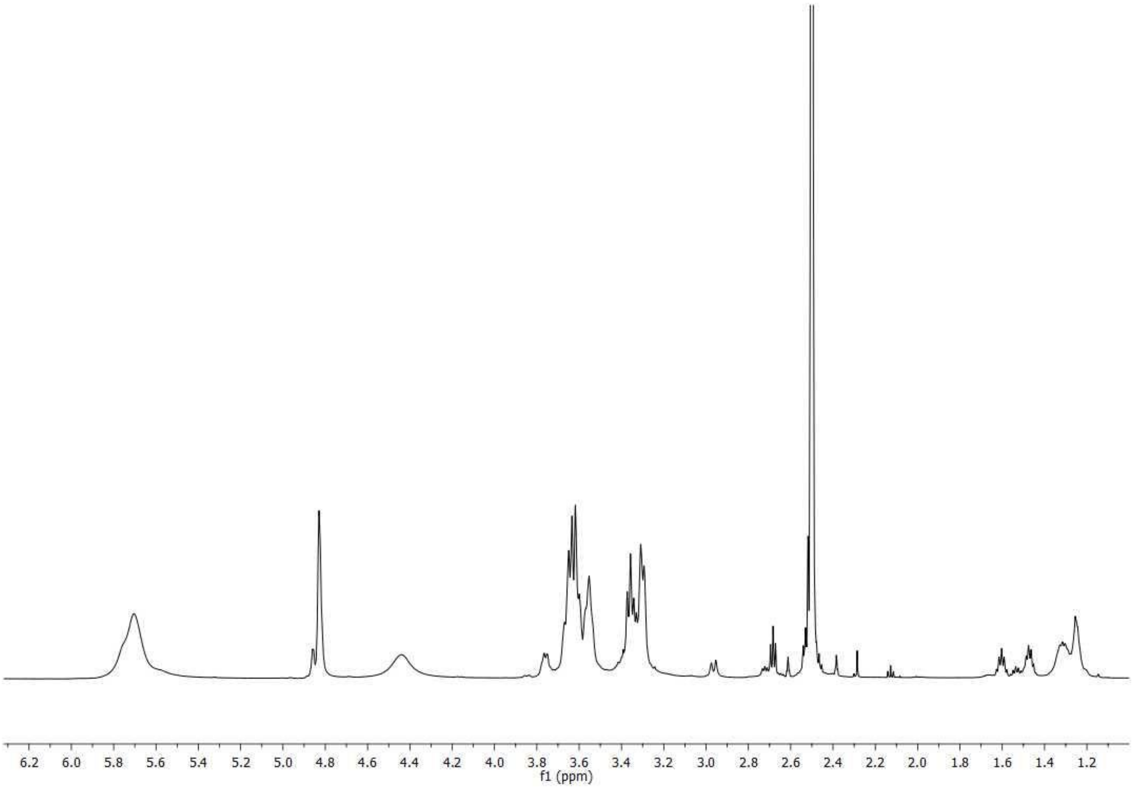

^{13}C

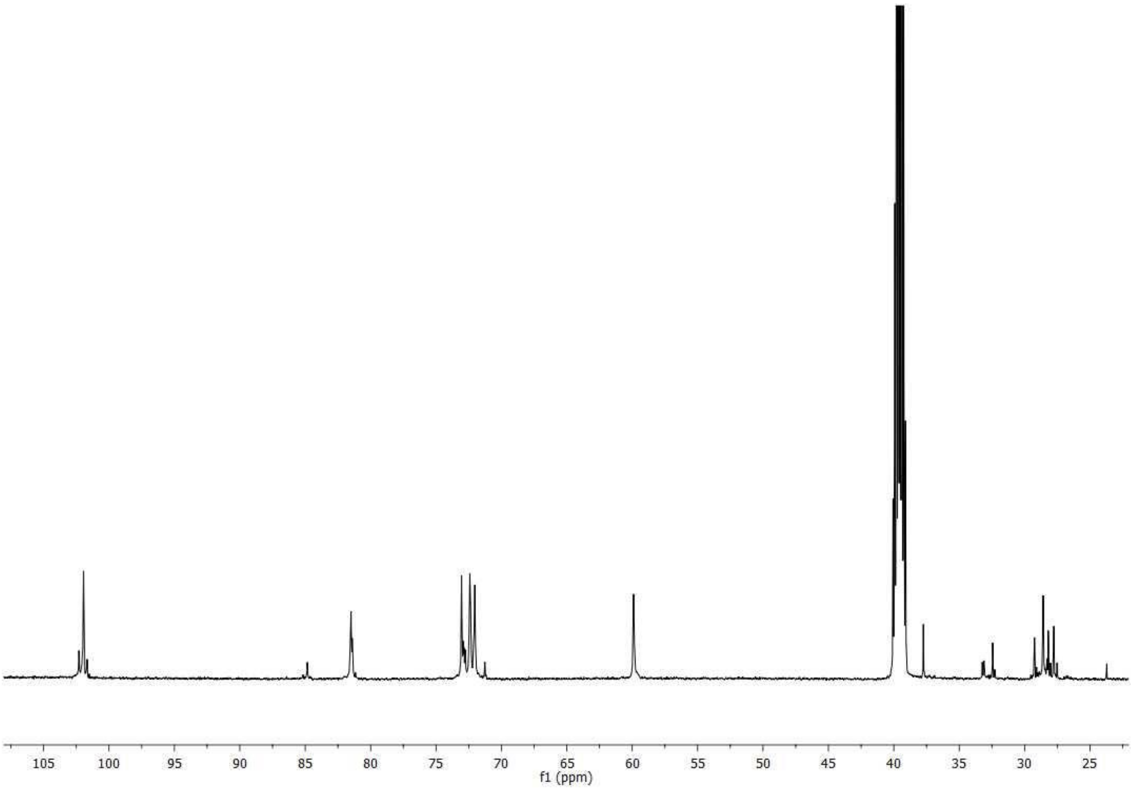

Disulfide 6a

^{1}H

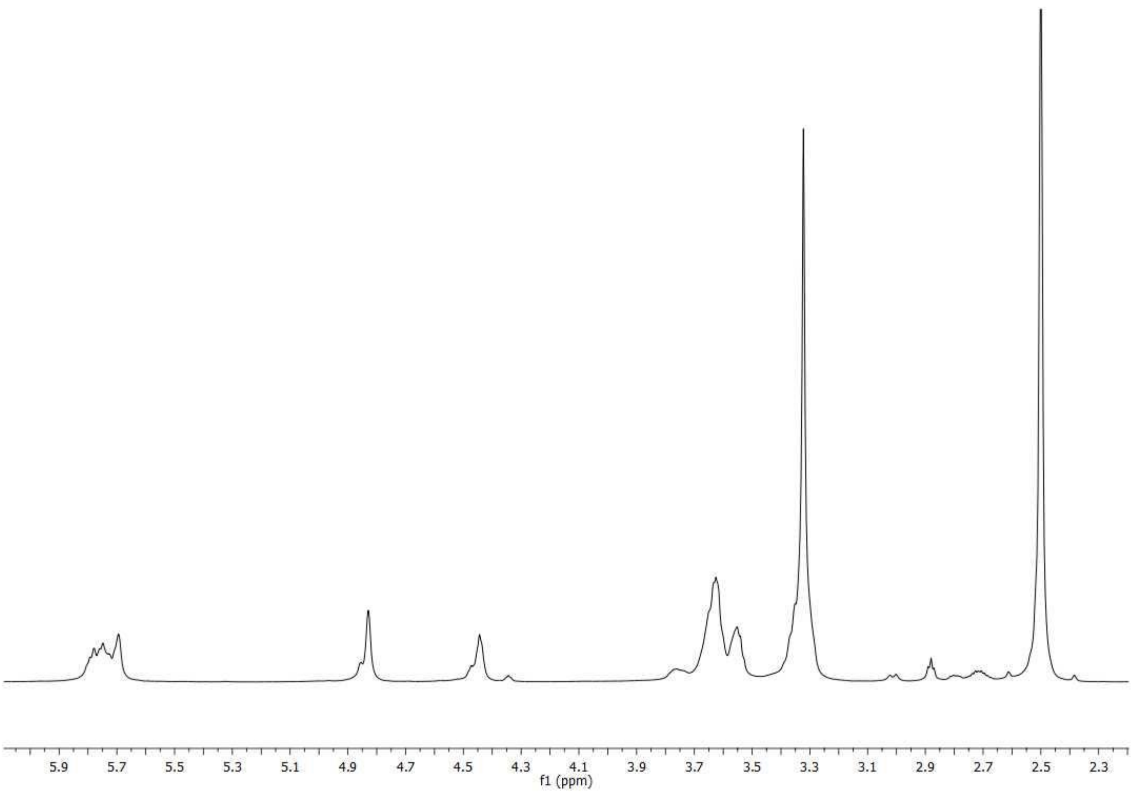

^{13}C

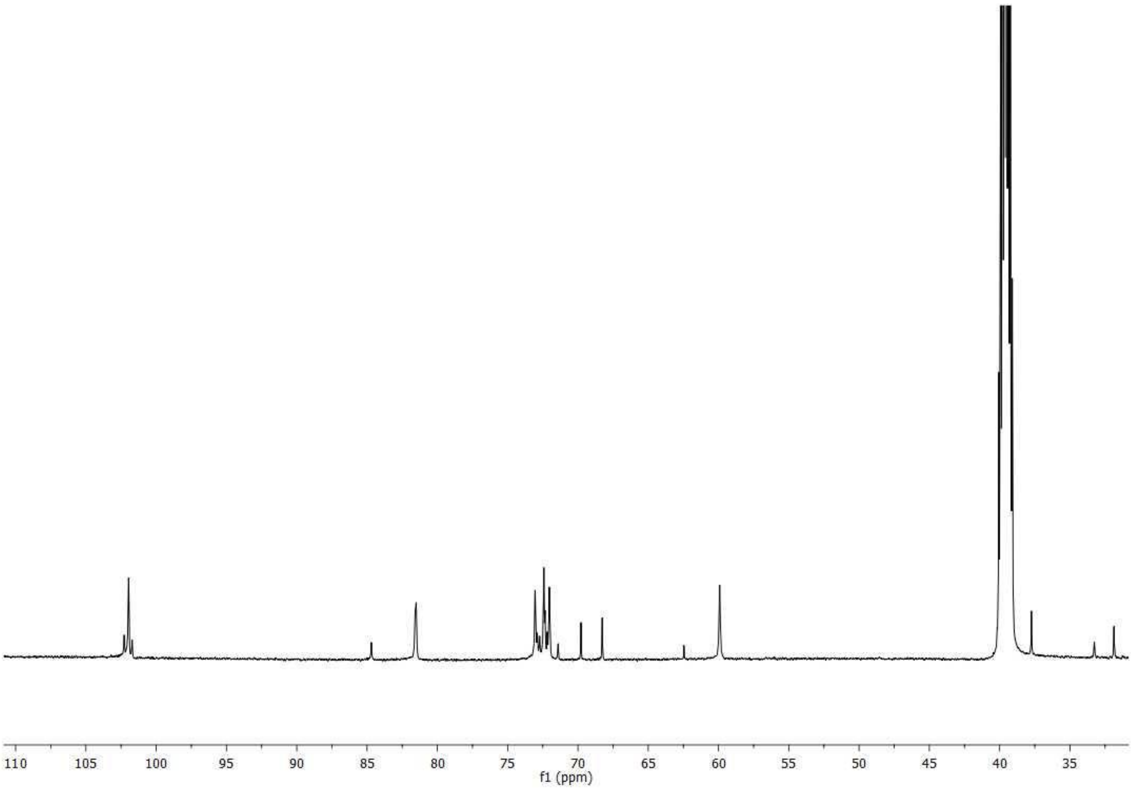

Disulfide 6b

^{1}H

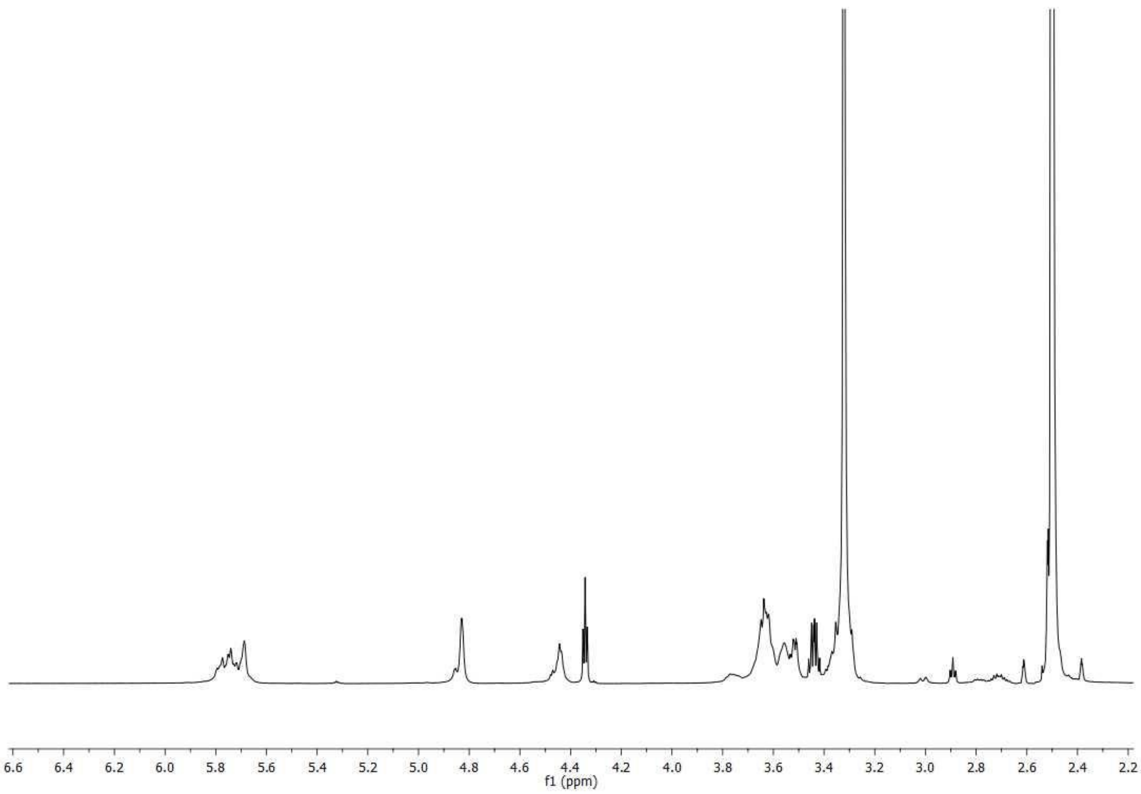

^{13}C

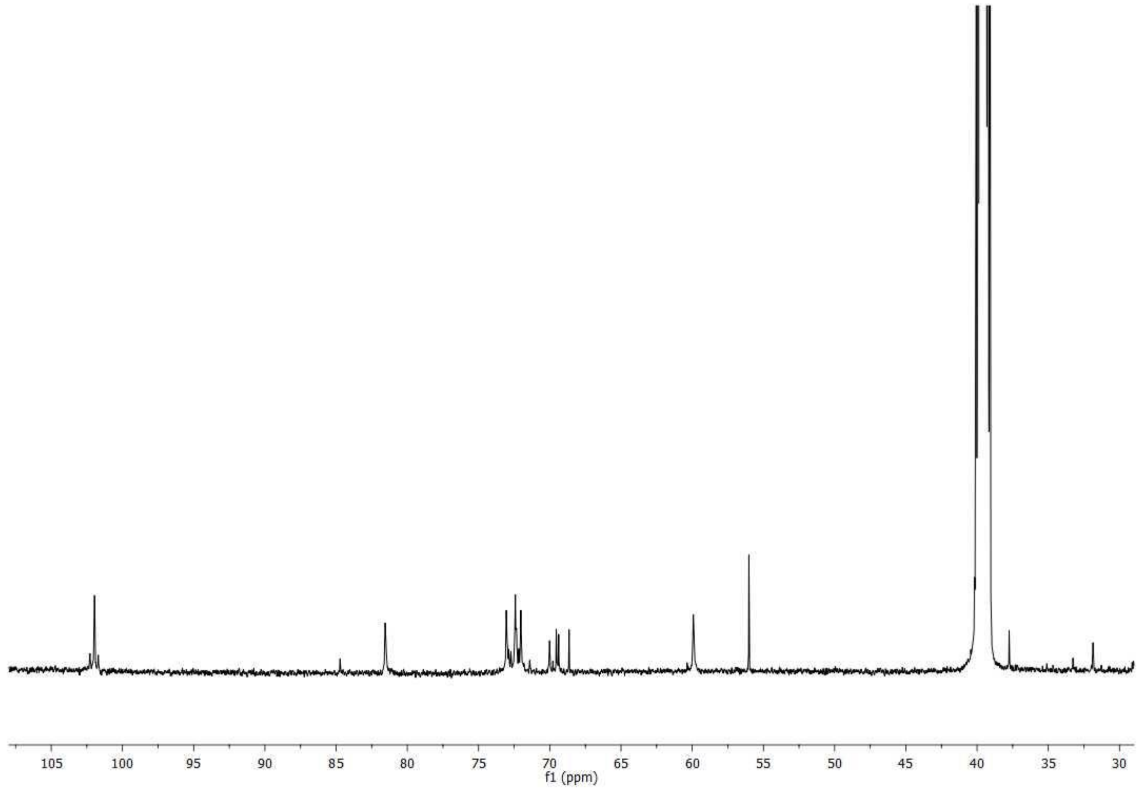

Disulfide 6c

^{1}H

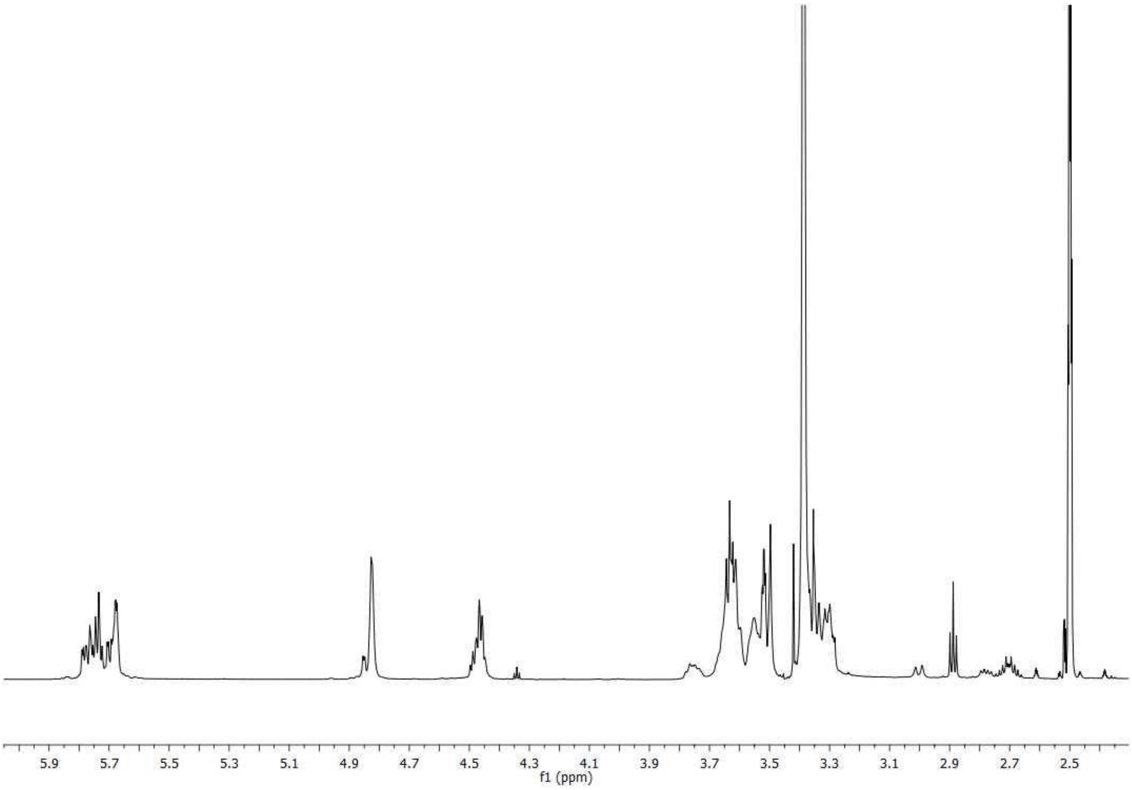

^{13}C

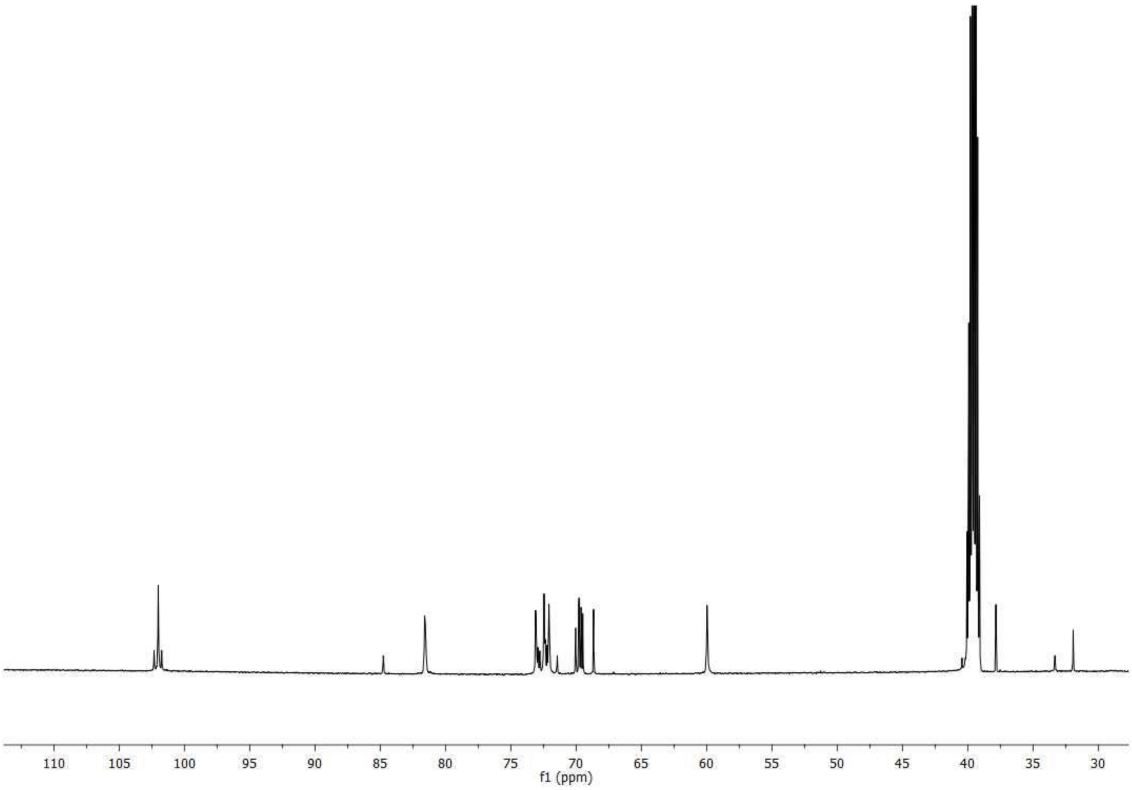

Disulfide 6d

^{1}H

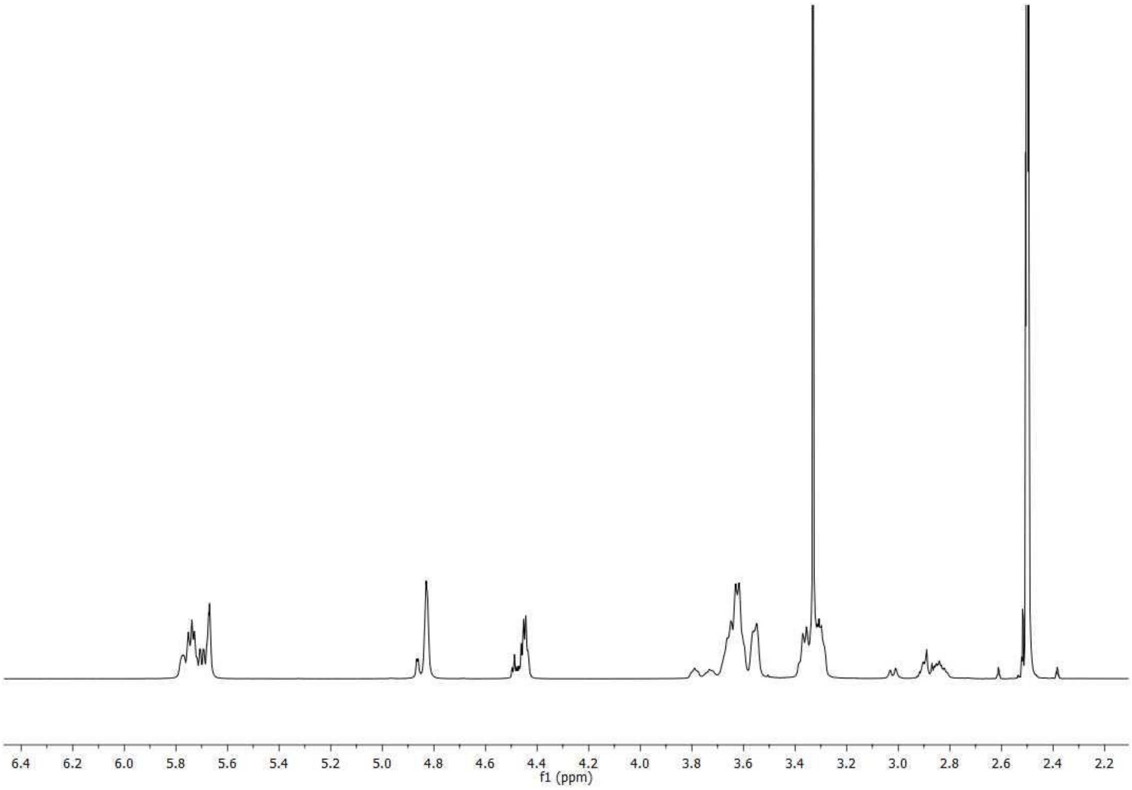

^{13}C

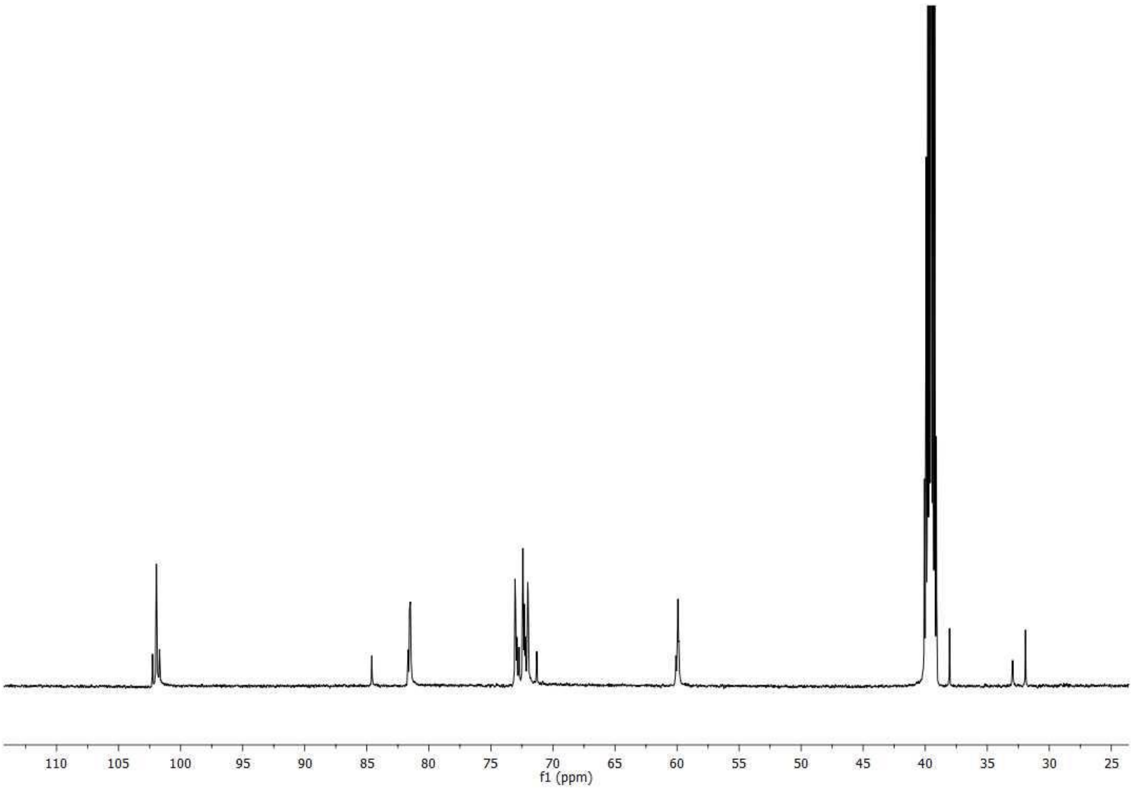

Disulfide 6e

^{1}H

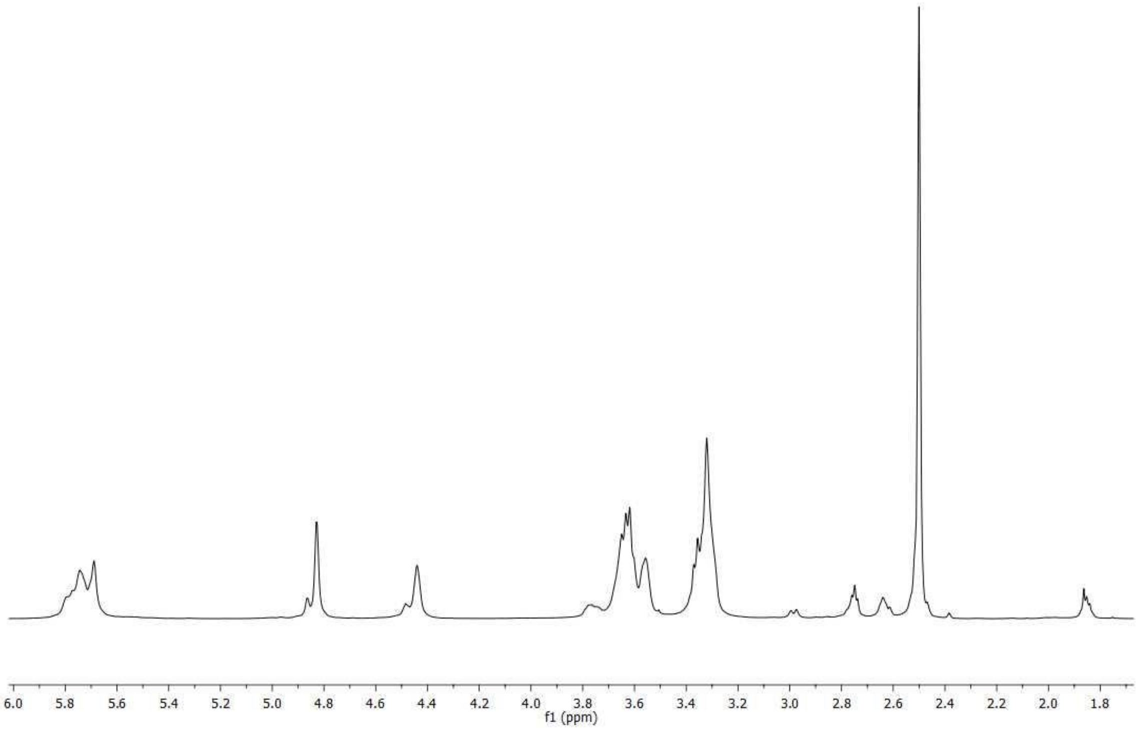

^{13}C

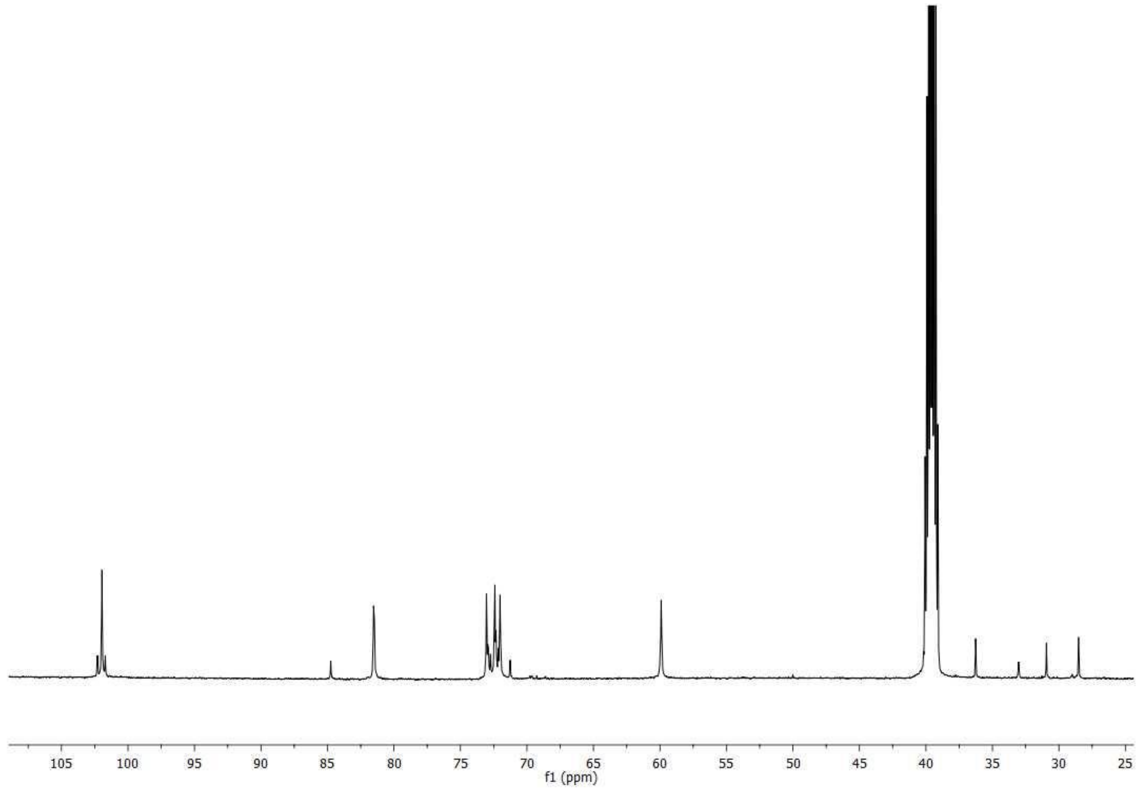

Disulfide 6f

^{1}H

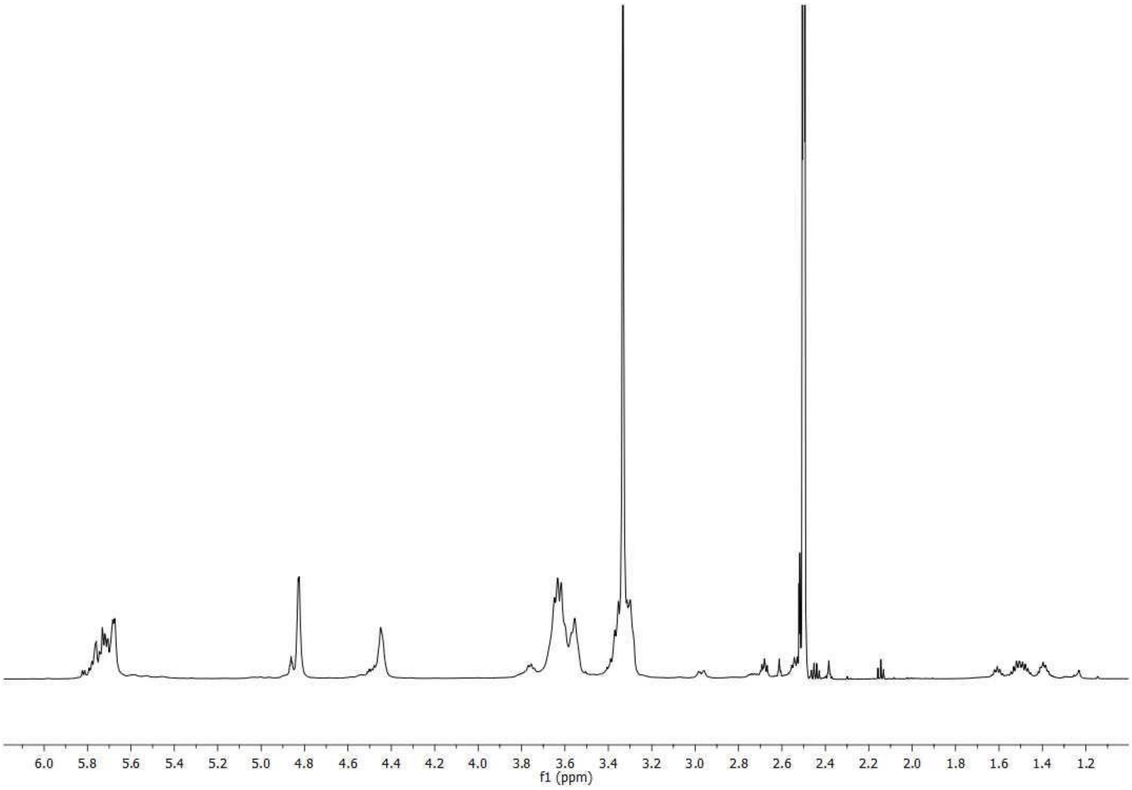

^{13}C

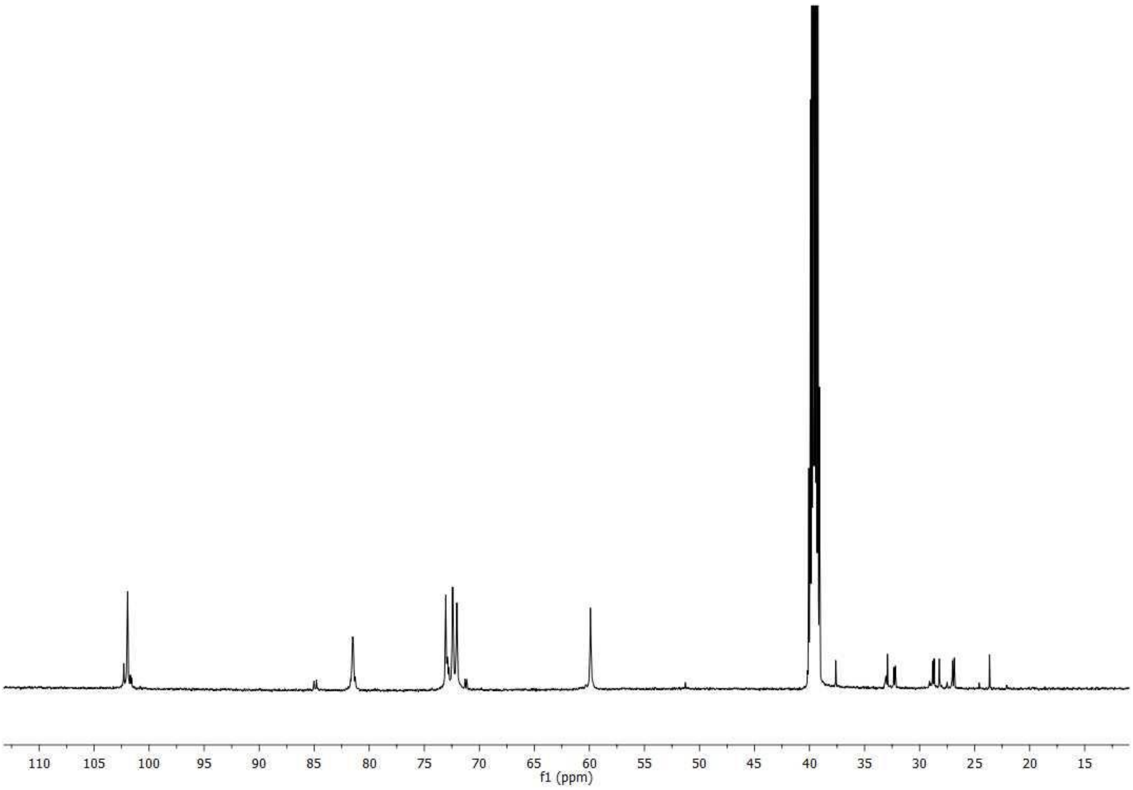

Disulfide 6g

^1H

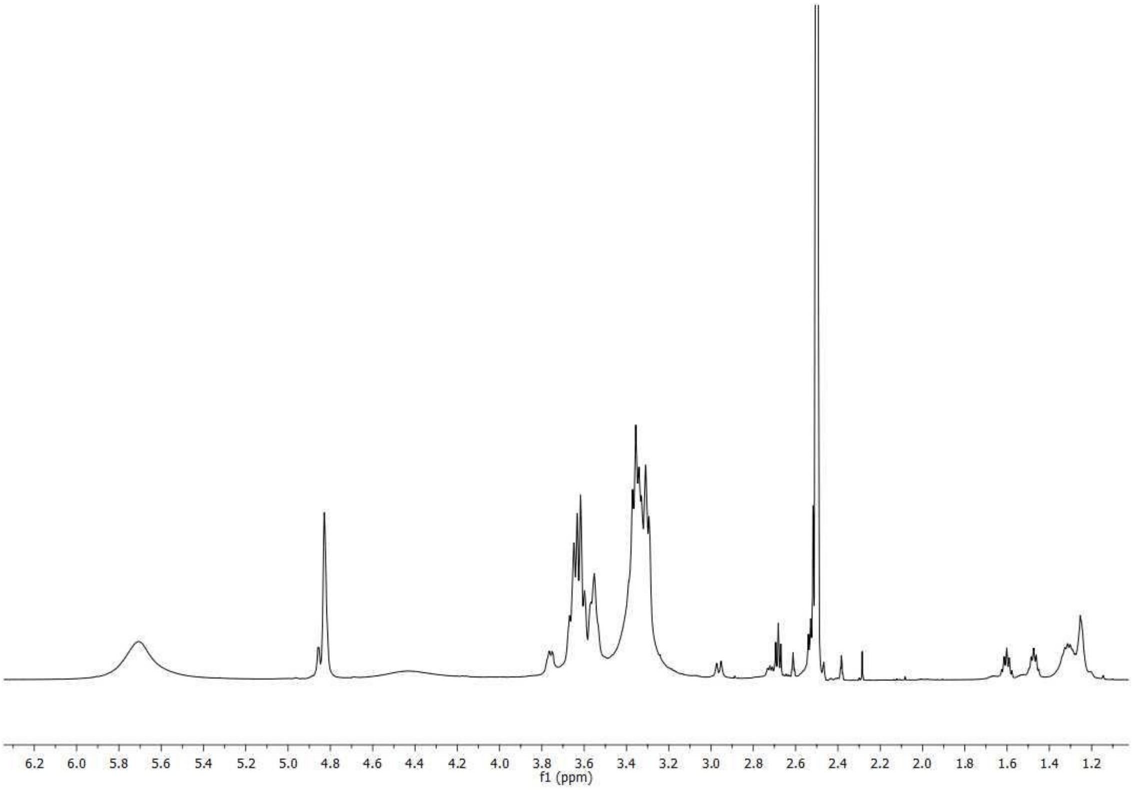

^{13}C

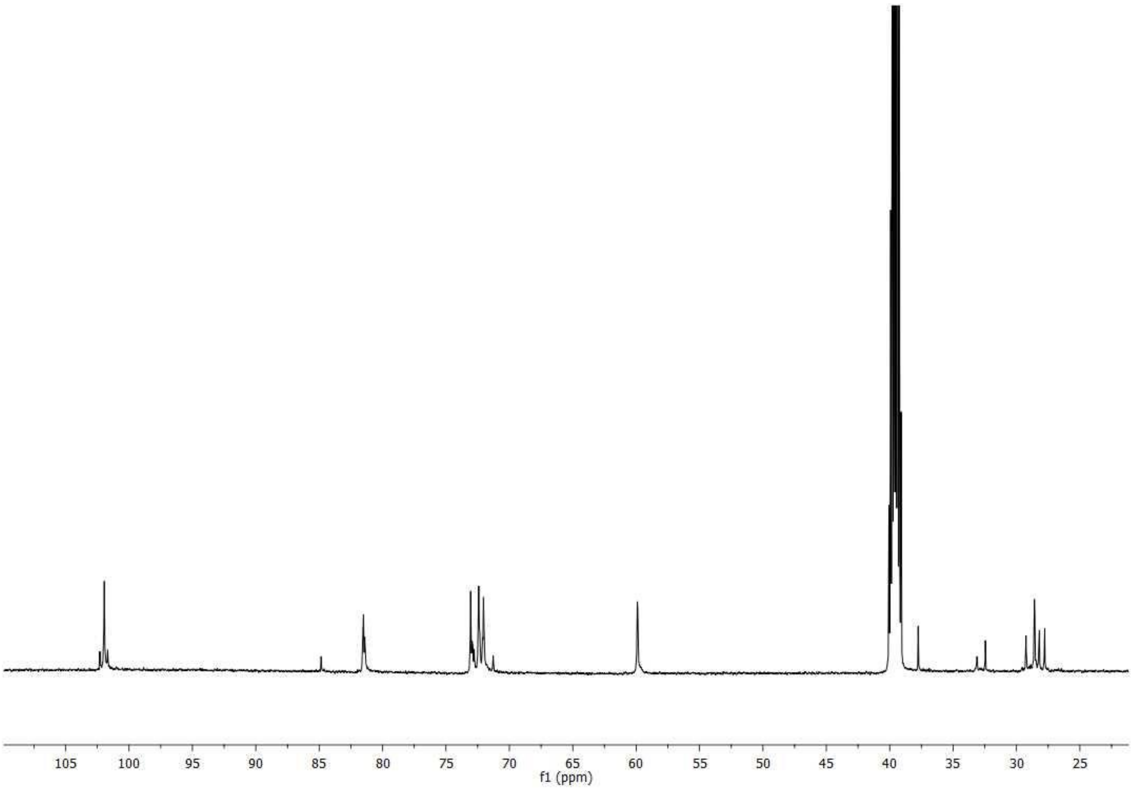

Supplement: File 1 — General experimental procedures, instruments, materials. Detailed experimental procedures and characterization data for newly prepared compounds. 1H and 13C NMR spectra of prepared compounds. [file Beilstein_J_Org_Chem-12-349-s001.pdf]
